# Social-interactive reward elicits similar neural response in autism and typical development and predicts future social experiences

**DOI:** 10.1016/j.dcn.2023.101197

**Published:** 2023-01-06

**Authors:** Kathryn A. McNaughton, Laura Anderson Kirby, Katherine Rice Warnell, Diana Alkire, Junaid S. Merchant, Dustin Moraczewski, Heather A. Yarger, Audrey Thurm, Elizabeth Redcay

**Affiliations:** a Neuroscience and Cognitive Science Program, University of Maryland College Park, USA; b Department of Psychology, University of Maryland College Park, USA; c Lauren Turner Brown PhD, PLLC, USA; d Department of Psychology, Texas State University, USA; e Division of Extramural Research, National Institute on Drug Abuse, USA; f Data Science and Sharing Team, National Institute of Mental Health, USA; g Office of the Clinical Director, National Institute of Mental Health, USA

**Keywords:** VS, Ventral Striatum, AUT, Autism, TD, Typical Development, Autism, Social interaction, Social reward, fMRI, Ventral striatum

## Abstract

Challenges in initiating and responding to social-interactive exchanges are a key diagnostic feature of autism spectrum disorder, yet investigations into the underlying neural mechanisms of social interaction have been hampered by reliance on non-interactive approaches. Using an innovative social-interactive neuroscience approach, we investigated differences between youth with autism and youth with typical development in neural response to a chat-based social-interactive reward, as well as factors such as age and self-reported social enjoyment that may account for heterogeneity in that response. We found minimal group differences in neural and behavioral response to social-interactive reward, and variation within both groups was related to self-reported social enjoyment during the task. Furthermore, neural sensitivity to social-interactive reward predicted future enjoyment of a face-to-face social interaction with a novel peer. These findings have important implications for understanding the nature of social reward and peer interactions in typical development as well as for future research informing social interactions in individuals on the autism spectrum.

## Highlights

• We used a social-interactive fMRI task to investigate social reward.
• We examined groups with autism (AUT) and typical development (TD).
• We found minimal differences between groups in social-interactive reward processing.
• Heterogeneity in neural response related to self-reported enjoyment of the task.
• Neural response predicted future social interaction enjoyment with a novel peer.

## 1 Introduction

Difficulties in social interaction, including challenges in initiating and responding to social exchanges, are central to the diagnosis of autism spectrum disorder (American Psychiatric Association, 2013). These challenges with social interaction may stem from atypical social motivation, including social reward processing (Dawson et al., 2005, Chevallier et al., 2012, Kohls et al., 2012). However, empirical support for differences in neural processing of social reward in autism is mixed, with results varying based on which reward construct and specific region of the reward network is studied (Bottini, 2018, Clements et al., 2018). This mixed evidence may arise from the artificial conditions (e.g., non-interactive lab-based tasks) that have conventionally been used to study social reward, or from heterogeneity in the neural processing of social reward in autism, or a combination of these two factors. Therefore, assessing neural processing of reward during naturalistic social interactions, examining factors that account for neural heterogeneity, and linking that heterogeneity to real-world social behavior is needed for a greater understanding of social reward processing and social interaction challenges in autism.

The neural circuitry involved in the anticipation and receipt of reward includes the ventral striatum (VS; containing the nucleus accumbens, ventral caudate, and ventral putamen) and orbitofrontal cortex, among other regions (Haber and Knutson, 2010, Berridge et al., 2009, Liu et al., 2011, Diekhof et al., 2012). While substantial work has characterized the involvement of these structures in non-social rewards such as food and money (Oldham et al., 2018), other research has highlighted the involvement of the VS in processing social feedback, such as smiling faces or positive social evaluations, suggesting a “common currency” within the VS for neural processing of reward that spans social and non-social rewards (Izuma et al., 2008, Spreckelmeyer et al., 2009, Ruff and Fehr, 2014).

The social motivation hypothesis argues that social reward processing in autism is reduced (Chevallier et al., 2012), yet neural and behavioral findings in support of this hypothesis have been mixed (Bottini, 2018, Clements et al., 2018, Jaswal and Akhtar, 2019). A few (though not most) functional neuroimaging studies on social reward in autism have identified hypoactivation of the VS to social rewards for individuals with autism compared to individuals with typical development, as well as reduced functional and structural connectivity between the VS and other regions of the reward network in response to social stimuli (Scott-Van Zeeland et al., 2010, Supekar et al., 2018). Yet others have failed to identify differences in VS activity between individuals on the autism spectrum and those with typical development in neural processing of social rewards (Dichter et al., 2012, Kohls et al., 2013, Delmonte et al., 2012). Consistent with mixed findings, a recent meta-analysis found no evidence for atypical VS response to social reward in autism (Clements et al., 2018). However, it did demonstrate small effects of hypoactivation within other regions associated with reward, including caudate and anterior cingulate, in response to rewarding stimuli in individuals with autism compared to individuals with typical development (Clements et al., 2018). Further, hypoactivation in regions associated with reward was not specific to social rewards. Thus, support for the social motivation hypothesis remains mixed.

One factor that could account for the mixed findings in prior studies of social reward processing in autism is the use of non-interactive rewards in studies. Non-interactive rewards (e.g., performing a button-press task to be rewarded with a static picture of a smiling face) may not be sufficient to evoke social reward as found in everyday interactions (Schilbach et al., 2013, Redcay and Schilbach, 2019). Use of social-interactive rewards is especially critical to understanding social reward in autism because interactive contexts may better elicit core social processing challenges (Rolison et al., 2015). Indeed, functional connectivity differences between individuals with autism and typical development are increasingly apparent in contexts with higher social demands, such as having a spontaneous conversation with an experimenter, compared to contexts with lower social demands, such as repeating an experimenter’s speech (Jasmin et al., 2019). Therefore, examining social reward in a social-interactive context provides an opportunity to better understand neural correlates of real-world social challenges for individuals on the autism spectrum.

A limited set of studies has examined social-interactive reward in individuals with typical development, primarily focusing on two aspects of real-world social interactions that engage reward network regions: sharing information about oneself and receiving responses from a partner. Tasks that have investigated these social-interactive rewards have identified VS activation in response to sharing information with a partner (Tamir and Mitchell, 2012, Tamir et al., 2015), as well as receiving engaged or positive responses from a peer via virtual text-based chat tasks (Warnell et al., 2018, Guyer et al., 2012, Gunther Moor et al., 2010, Silk et al., 2014). In addition to engaging the reward network, social-interactive exchanges are further characterized by increased engagement of the social-cognitive network, including temporal regions such as the temporoparietal junction (TPJ), superior temporal sulcus, and anterior temporal lobe; frontal regions such as the dorsomedial and ventromedial prefrontal cortex; and precuneus (Warnell et al., 2018, Alkire et al., 2018). The engagement of both social-cognitive and reward networks highlights how social-interactive contexts tend to engage broader networks of brain activity, particularly regions related to reward and social-cognitive processing, compared to non-interactive contexts (Redcay and Schilbach, 2019). To date, however, these social-interactive paradigms have not been extended to autism, and thus the neural correlates of social-interactive reward in autism are unknown.

In addition to non-interactive paradigms, another factor that could account for the mixed results of prior studies of reward processing in individuals on the autism spectrum is heterogeneity in reward processing. Autism is a highly heterogeneous disorder, and aspects of social motivation may differ across individuals (Neuhaus et al., 2021). Notably, many individuals on the autism spectrum report an interest in social interaction and forming relationships (Jaswal and Akhtar, 2019). This heterogeneity could result in contradictory findings across studies, particularly given that most studies have employed small sample sizes. Therefore, it is important to consider social reward processing in the context of individual differences in self-reported social motivation, which may account for variation in social reward processing circuitry. Furthermore, the neural processing of social rewards may vary by age and autism severity (Clements et al., 2018). As heterogeneity in symptom severity, age, and self-reported social motivation in samples may contribute to the mixed findings of atypical social reward processing in autism (Bottini, 2018, Clements et al., 2018), assessing the relationship between social reward processing and individual difference factors could clarify these mixed results.

Characterizing social reward processing in autism is particularly important during middle childhood and early adolescence (8–14 years old). In typical development, middle childhood is a period in which peer relationships become increasingly important (Ladd, 1999), and the neural circuitry for social-cognitive processing becomes increasingly specialized (Gweon et al., 2012, Redcay and Warnell, 2018). However, it is also a time in which differences in social-cognitive task performance (Bal et al., 2013) and social communication (Wallace et al., 2017) may become greater between youth on the autism spectrum and their peers with typical development. Characterizing the neural correlates of social reward processing in autism will provide insight into reward system functioning during this crucial developmental period.

Beyond simply identifying neural correlates of social reward in typical development and autism, it is important to connect neuroimaging findings to behavior in the real world. Neural response to social reward predicts real-world enjoyment of established peer relationships for typically developing adolescents (Alarcón et al., 2020, Flores et al., 2018). Additionally, friendship stability in typically developing adolescents predicts reward network response to rewards given to friends (Schreuders et al., 2021). Although these findings provide evidence of the importance of neural responsivity to social reward in predicting real-world behavior, it is unknown how neural processing of social reward in autism relates to real-world social interaction, which would provide a critical link between reward network activity and social interaction challenges in autism.

To address these gaps in the literature, we examined the neural correlates of social reward in a chat-based interactive context during middle childhood and early adolescence. Specifically, we investigated the neural correlates of social-interactive reward, both sharing self-relevant information and receiving engaged responses, in youth with and without autism as well as differences between groups. We predicted greater engagement of key reward regions (e.g., VS) to peer, compared to computer, conditions but a reduced difference between peer and computer conditions in autism. To test these hypotheses, we examined neural response to social-interactive reward both within and between groups in whole-brain analyses and region-of-interest analyses focusing on the VS. We further predicted that self-reported social motivation and age would account for heterogeneity in VS response to interactive social reward in youth generally. Finally, we predicted that VS response to social-interactive reward would predict subjective responses to a later social interaction, specifically self-reported enjoyment of a face-to-face, getting-to-know-you interaction with an unfamiliar peer.

## 2 Methods

### 2.1 Participants

Youth with typical development and youth with autism were recruited from fliers, outreach events, the Interactive Autism Network, Simons Foundation Powering Autism Research (SPARK), and a database of local families interested in participating in research. We appreciate obtaining access to recruit participants through SPARK research match on SFARI Base. Inclusion criteria required full-term, native English speakers with no known history of head injury or seizures and no contraindications for MRI, and IQ scores above 80 as assessed by the Kaufman Brief Intelligence Test, Second Edition (KBIT-2 (Kaufman and Kaufman, 2004)). Inclusion in the typical development (TD) group (*n* = 99) required no history of psychiatric disorders and no first-degree relatives with autism or schizophrenia. Inclusion in the autism (AUT) group (*n* = 62) required a previous diagnosis of autism spectrum disorder and scores above the ‘autism spectrum’ cut-off (above 7) from administration of Module 3 of the Autism Diagnostic Observation Schedule, 2nd edition (ADOS-2 (Lord et al., 2012)) by a research-reliable administrator as part of the study. All procedures were approved by the University of Maryland Institutional Review Board, and parents and youth provided informed consent and assent.

Data from participating individuals was excluded as follows. Eight youth (*n* = 5 AUT, *n* = 3 TD) began but did not complete the structural and functional scans of the MRI protocol, one youth (*n* = 1 TD) was excluded for an abnormal clinical finding in the scan, 21 youth (*n* = 12 AUT, *n* = 9 TD) were excluded for excessive scan motion (see Section 2.5), and 17 youth (*n* = 2 AUT, *n* = 15 TD) were excluded for not believing they were chatting with a real peer (see Section 2.3.3). There was a marginal difference such that youth in the AUT group who were excluded from analysis had *less* severe symptoms (M=9.6 on ADOS-2 Module 3 Overall Total) compared to those included (M = 11.0 on ADOS-2 Module 3 Overall Total) (*t*(60) = − 1.70, *p* = 0.09).

After these exclusions, the final sample included in analyses here consisted of 114 youth (*n* = 43 AUT, *n* = 71 TD). Race, ethnicity, and household income data for the full sample of 114 youth are presented in Supplemental Table 1.

A subsample of 43 TD youth was selected from the full TD sample (*n* = 71) to be a gender-, mean age-, and mean IQ-matched group to the AUT group (‘Matched Sample’) for analyses. The full sample of 71 youth in the TD group was used for individual differences analyses to increase statistical power (Table 1). Data on a subset of the youth in the TD group (*n* = 22 used in the full sample, *n* = 12 used in the matched sample) were reported in a previous set of analyses (Warnell et al., 2018).

**Table 1:** Sample demographics.

Group – Matched Sample	AUT	Matched TD
*n*	43	43
Gender	36 male, 7 female	36 male, 7 female
Mean age (range)	11.84 (7.58–14.95)	11.63 (8.10–14.71)
Mean KBIT-2 (range)	113 (86–141)	116 (83–146)
Mean ADOS-2 Module 3 Overall Total (SA + RRB) (range)	11 (7–20)	NA
		
Group – Full Sample	AUT	Full TD
*n*	43	71
Gender	36 male, 7 female	43 male, 28 female
Mean age (range)	11.84 (7.58–14.95)	11.11 (8.10–14.71)
Mean KBIT-2 (range)	113 (86–141)	118 (83–146)
Mean ADOS-2 Module 3 Overall Total (SA + RRB) (range)	11 (7–20)	NA
		
Group – Longitudinal, Completed Interaction Quality	AUT	TD
*n*	23	48
Gender	19 male, 4 female	27 male, 21 female
Mean age at follow-up (range)	13.77 (10.23–16.17)	12.81 (9.36–16.05)
Mean KBIT-2 (range)	116 (86–141)	121 (87–146)
Mean ADOS-2 Module 3 Overall Total (SA + RRB) (range)	11 (7–16)	NA
		
Group – Longitudinal, Completed Desire to Interact Again	AUT	TD
*n*	15	43
Gender	11 male, 4 female	23 male, 20 female
Mean age at follow-up (range)	13.59 (10.23–16.17)	12.63 (9.36–15.90)
Mean KBIT-2 (range)	116 (95–134)	121 (87–142)
Mean ADOS-2 Module 3 Overall Total (SA + RRB) (range)	11 (8–16)	NA

### 2.2 Behavioral tasks and self-report measures

Youth were initially recruited into a study of social processing in typical development and autism, which consisted of two MRI visits and a collection of behavioral tasks. Details of one of the MRI scans are provided here (see Section 2.3), while details of the other MRI scan that a subset of participants completed have been previously reported (Alkire et al., 2018). Families were later re-contacted to take part in a follow-up study (0.6–4.5 years later, mean = 1.9 yrs, SD =1.1 yrs), which consisted of a peer interaction task and an additional selection of behavioral tasks (see Section 2.2.1), a subset of which are reported here.

#### 2.2.1 Follow-up behavioral visit

At the follow-up behavioral visit, participants completed a 25-min social interaction with a novel peer who was also a participant. Pairs were matched for gender and within a year of chronological age. Participants engaged in five minutes of free conversation, a fifteen-minute list-making task, and a five-minute video-watching task, a structure similar to other developmental social interaction tasks used to investigate live social interaction with a novel peer in autism (Usher et al., 2015). Following the interaction, participants completed two measures designed to assess interaction enjoyment. One measure was a six-item questionnaire adapted from other questionnaires used to measure interaction quality in autism and typical development (Morrison et al., 2020, Berry and Hansen, 1996). The second measure was a single slider-response question (− 100 to 100 with 1-point increments) designed to assess their desire to interact with the same partner again (see Supplemental information). Both of these measures reflect enjoyment of the peer interaction, and were therefore considered measures of interaction enjoyment.

Of the 114 participants, 73 returned to participate in the peer interaction visit (*n* = 24 AUT). One participant was excluded from the analyses described in this paper because they were previously familiar with their peer interaction partner. One participant did not complete the six-item questionnaire of interaction quality, and 14 participants did not complete the slider response question (see Table 1 for demographics).

Of the 71 participants that completed the interaction quality questionnaire, 38 were participants in the TD group paired with participants in the TD group, 10 were participants in the TD group paired with participants in the AUT group, 16 were participants in the AUT group paired with participants in the TD group, and 7 were participants in the AUT group paired with participants in the AUT group. Participants whose partners were in the AUT group did not differ in rating of interaction quality compared to participants whose partners were in the TD group (*t*(69) = 0.77, *p* = 0.46).

### 2.3 FMRI task

#### 2.3.1 Task design

**Fig. 1:**
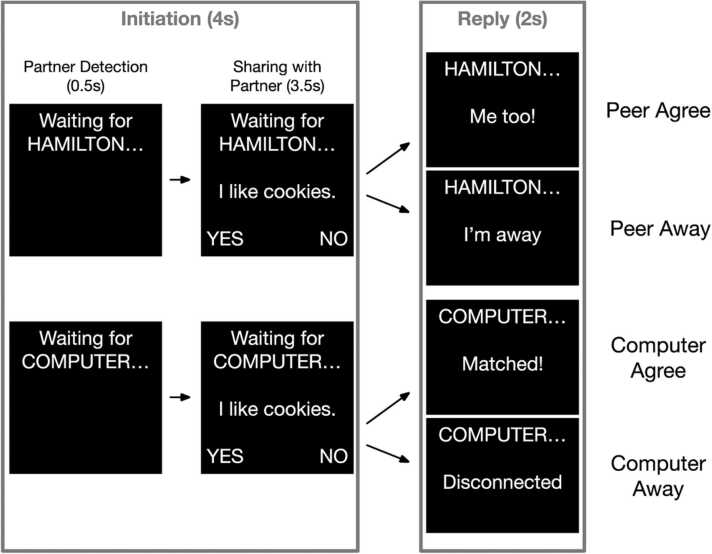
Social-interactive reward task design. During the initiation period, participants sent messages about their interests (e.g., “I like cookies”) to a perceived peer or a computer partner. During the reply period, participants received engaged (e.g., “Me too!”/“Matched!”) or disengaged (e.g., “I’m away”/“Disconnected”) responses.

Youth participated in a real-time peer interactive chat task described previously (Warnell et al., 2018). Briefly, youth were informed that they would be chatting with a gender- and age-matched partner in another lab even though all interaction was simulated. Outside of the scanner, youth were shown photos and names of two age and gender-matched peers. Youth were then given the opportunity to choose their chat partner (Supplemental Fig. 1). To make the peer illusion believable, youth also had their pictures taken and were told that their interaction partner could see their picture. In the scanner, the participants viewed “messages” via a projector screen and could send messages about their interests (e.g., I like cookies) by using buttons to respond “yes” or “no” to statements about their likes and dislikes (Initiation Period; see Fig. 1 for task structure). The participants were instructed that their partner could sometimes see the participant’s message and respond with an engaged agree or disagree message (“Me too!”/“That’s not what I picked”) (Reply Period). Participants were instructed that at other times, their partner would see the participant’s message but would be unable to respond because the partner was required to play a different game. On these trials, the participant would see the peer’s response as an unengaged away message (“I’m away”). In addition to chatting with the peer, youth were also informed that they would sometimes chat with a computer that would respond to their messages with an engaged response that would be randomly selected to match or not match the participants’ response (“Matched!”/“Mismatched”). Alternatively, participants were informed the computer may become disconnected and send an unengaged response (“Disconnected”). The task followed a 2 × 2 design in which the effects of Partner (Peer/Computer) and Engagement (Agree/Away) could be simultaneously examined (Fig. 1). Following this explanation, youth were asked comprehension questions to ensure they understood the task.

#### 2.3.2 Stimuli

As described previously (Warnell et al., 2018), participants viewed 104 trials (52 peer, 52 computer) in 4 runs of 26 trials each. Each run began with an approximately 15 s fixation cross and ended with an approximately 10 s fixation cross.

Each trial consisted of an initiation phase (4 s) and a reply phase (2 s) (Fig. 1). In between the initiation and reply phase, and in between each trial, youth viewed a 2–6 s jittered fixation cross (mean of 3.5 s and exponentially distributed).

In the initiation phase, the participant saw whether they were chatting with the peer or the computer (0.5 s). Next, the participant responded yes or no to a statement about their interests (3.5 s). Reaction time was calculated as the response latency to this statement. If the participant did not respond within the 3.5 s response window in the initiation phase, they saw a non-engaged reply, and these trials were excluded from analysis as skipped trials. Runs were excluded if participants skipped more than eight trials in a run (1/3 of trials in a run). Three participants had runs excluded for meeting this criterion. Participants were to be excluded if they skipped more than half of trials of a given condition across all four runs. No participants were excluded for meeting this threshold.

In the reply phase, participants received an engaged or disengaged message from their partner for that trial. Participants received responses evenly divided between peer engagement (“Me too!”), peer non-engagement (“I’m away”), computer engagement (“Matched!”), and computer non-engagement (“Disconnected”), resulting in 24 responses of each type. To enhance the illusion that they were interacting with an actual peer, participants also received 2 disagree responses from the peer (“That’s not what I picked”) and 2 from the computer (“Mismatched”) in each run, which were not analyzed.

#### 2.3.3 Post-test

Following completion of the scan, an experimenter verbally administered a post-test to the participants to assess their self-reported enjoyment of interacting with the peer and computer partners, their attention to the peer and computer answers, and their desire to see the peer and computer answers (Warnell et al., 2018). The complete post-test is provided as Supplemental material. Youth were debriefed by a member of the research team, or parents were provided with debriefing materials following completion of the study. Participants’ responses to the post-test questions assessing belief in the peer illusion (i.e. Was ____ a real person? (if no, why do you think that?) Do you think there was more to this game than we told you? (if yes, what?) Is there anything else you want to tell us about the chat task?) were used alongside experimenter and parent judgment of their child’s belief (following debriefing) to determine the participant’s belief in the peer illusion. As noted earlier, seventeen youth expressed disbelief in the peer illusion and were excluded from further analysis.

### 2.4 Behavioral data analysis

#### 2.4.1 Scanner task performance

To compare scanner task performance between the AUT and TD groups, the matched sample (*n* = 86) was used. To examine differences in reaction time between groups and across the peer and computer partner conditions of the scanner task, a 2 (Partner: Peer/Computer) × 2 (Group: TD/AUT) ANOVA was run on the individuals’ median reaction time for the trials in each condition. A 2 (Partner: Peer/Computer) × 2 (Group: TD/AUT) ANOVA was also run on the proportion of skipped statements. Statistical analyses were run in R Core Team (2018). Means and standard deviations by group for behavioral data are provided in Supplemental Table 2.

#### 2.4.2 Post-test

Responses to two pairs of single questions on the post-test were analyzed using non-parametric analyses (Wilcoxon Sign Rank) to compare preference for peer and computer partner within groups (‘How much did you like chatting with …’, ‘How much did you want to see ____ answer…’). Multiple items that corresponded to enjoyment of the peer partner and chatting were summed to form a ‘peer interaction enjoyment composite’ (see Supplemental material), which was compared between TD and AUT groups using an independent samples two-sided *t*-test.

### 2.5 Image acquisition and preprocessing

fMRI data were collected on a Siemens 3T scanner using a 32-channel head coil (MAGNETOM Trio Tim System, Siemens Medical Solutions). The scanning protocol included four runs of functional MRI data acquisition (T2*-weighted gradient echo-planer images, 40 interleaved axial slices, voxel size = 3.0 × 3.0 × 3.3 mm, repetition time = 2200 ms, echo time = 24 ms, flip angle = 78°, pixel matrix = 64 × 64, 168 or 170 volumes due to addition of volumes in scanning protocol midway through study) and 1 structural scan (three-dimensional T1 magnetization-prepared rapid gradient-echo sequence; 192 contiguous sagittal slices, voxel size = 0.45 × 0.45 × 0.9 mm; repetition time = 1900 ms; echo time = 2.32 ms; flip angle = 9°; pixel matrix = 512 × 512).

Preprocessing of fMRI data was conducted using fMRIPrep version 1.4.1 (Esteban et al., 2019). Each participant’s anatomical image was segmented and normalized to MNI space (MNI Pediatric template, cohort 4: 7.5–13.5 years (Fonov et al., 2011)). Participants’ functional images were corrected for susceptibility distortion, realigned, and slice-time corrected. Next, functional images were co-registered, warped to the normalized anatomical image, and upsampled to 2 mm isotropic to match the normalized anatomical image. Functional data were smoothed with an isotropic 6 mm full-width half-maximum Gaussian kernel, and independent components analysis (ICA-AROMA) (Pruim et al., 2015) was used to remove motion artifacts in the data. Functional data were intensity normalized to a mean of 100 per voxel using 3dcalc in AFNI (Cox and Hyde, 1997, Cox, 1996). Additional details on the preprocessing pipeline are available in the supplemental information.

Runs with greater than 0.5 mm mean frame displacement across the entire run were excluded. Volumes with greater than 1 mm of displacement between volumes were censored (see Section 2.6.1). Runs with more than 20 % of censored volumes were excluded. Participants with less than three useable runs were excluded in further analyses (*n* = 21). 16 participants (*n* = 9 AUT) were included with three useable runs, and 98 participants (*n* = 34 AUT) were included with four useable runs.

### 2.6 fMRI data analysis

Following preprocessing, fMRI data were analyzed at both the whole-brain and region-of-interest (ROI) level. ROI analyses were performed with two VS ROIs. These ROIs were chosen based on previous evidence for a role of VS activation in social reward processing differences in autism (Clements et al., 2018) and in social-interactive reward in typical development (Warnell et al., 2018). The two VS ROIS (inferior VS corresponding to nucleus accumbens and superior VS corresponding to ventral caudate) were chosen from previous work on VS connectivity (Di Martino et al., 2011). An anatomically defined amygdala ROI was also investigated given the role of amygdala activation in social reward processing differences in autism and social-interactive reward in typical development, and these results are presented in the Supplemental information.

#### 2.6.1 First-level analyses

General linear models were constructed using the 3dREMLfit command in AFNI (Cox and Hyde, 1997, Cox, 1996). Events of interest (Peer Initiation, Computer Initiation, Peer Agree, Peer Away, Computer Agree, Computer Away) and events of no interest (Disagree responses, as well as Initiations and Reply events in trials in which the participant did not provide a response) were convolved with the canonical hemodynamic response function. To account for the fact that slice-time correction in fMRIPrep defaults to the middle of the volume, TR/2 = 1.1 s were subtracted from the stimulus times for these events. Six motion parameters (x, y, z, roll, pitch, yaw), their derivatives, and time points censored due to motion over 1 mm were also included as regressors (Power et al., 2014). Finally, constant, linear, quadratic, and cubic terms were also included to account for baseline and drift.

A total of six contrasts were created to test hypotheses about neural response to sharing self-relevant information and receiving reciprocal responses. In the initiation phase, the effect of Partner (Peer Initiation vs. Computer Initiation) was tested. In the reply phase, the main effect of Partner ([Peer Agree + Peer Away) vs. (Computer Agree + Computer Away)], main effect of Engagement [(Peer Agree + Computer Agree) vs. (Peer Away + Computer Away)], and their interaction were tested. Additionally, pairwise comparisons were examined to isolate specific effects related to social-interactive reward: the effect of engagement in social contexts [Peer Agree vs. Peer Away] and the effect of social context with engaged responses [Peer Agree vs. Computer Agree]. The two pairwise comparisons, effect of social context without engaged responses [Peer Away vs. Computer Away] and effect of engagement in non-social context [Computer Agree vs. Computer Away] are provided in Supplemental information Figs. 2 and 3.

#### 2.6.2 Second-level analyses

##### 2.6.2.1 Group effects

To test the hypothesis that the neural correlates of social-interactive reward differ between youth with autism and youth with typical development, whole-brain comparisons were performed with mixed-effects multilevel analysis using 3dMEMA in AFNI (Chen et al., 2012) on the matched TD-AUT sample (*n* = 86). These analyses were performed to compare AUT and TD group maps and to look within group maps to identify regions sensitive to social interaction. Analyses were masked with the intersection of the participant-level whole-brain masks. Age and mean FD were included as covariates in both between-group and within-group analyses. Contrast maps were thresholded at voxel-wise *p* < 0.001 and cluster-corrected at alpha = 0.05 (k = 126–141, bi-sided, second nearest-neighbor). Cluster correction values were calculated for each contrast using the –Clustsim option in AFNI’s 3dttest++ (Cox et al., 2017).

To further test the hypothesis of group differences between youth with autism and youth with typical development in neural response to social-interactive reward, group differences in activation in the two ROIs were examined in the matched sample (*n* = 86). Beta values were extracted for the Reply period from the bilateral regions of interest, and a 2 (Group: AUT/TD) × 2 (Partner: Peer/Computer) × 2 (Engagement: Agree/Away) ANOVA was run for each ROI in R Core Team (2018). Follow-up Bayesian analyses quantifying the strength of evidence against group effects were run in JASP (JASP Team, 2020) and interpreted using published guidelines (van Doorn et al., 2021).

##### 2.6.2.2 Individual differences

To examine factors that may account for heterogeneity in neural response to interactive social reward among youth with autism and youth with typical development, linear regressions were performed to predict neural sensitivity to social interaction in the VS ROIs in the full sample (*n* = 114) with age or self-reported enjoyment of the peer in the scanner task as predictors. Neural sensitivity to social-interactive reward in the VS ROIs was operationalized as the interaction term calculated from the extracted beta values from each ROI for the reply period [Peer Agree – Peer Away] – [Computer Agree – Computer Away].

Separate linear regressions were run in R to predict neural sensitivity to social interaction in the VS from each of the hypothesized predictors: (1) a composite of self-reported enjoyment of the peer during the fMRI task (see Section 2.3.3) and (2) age, for a total of 4 regression models. Self-reported enjoyment of the peer in the fMRI task and age were also tested as significant predictors of variation in amygdala response to social-interactive reward, and these analyses are reported in the Supplemental information.

##### 2.6.2.3 Predicting social interaction

Additionally, to test the hypothesis that neural sensitivity to social interaction may predict later real-world social interaction enjoyment, neural sensitivity to social interaction in the VS ROIs (i.e., the interaction term) was also used to predict the two measures of self-reported experience of enjoyment in a face-to-face live social interaction with a novel peer. Linear regressions were run in R to predict (1) the interaction quality composite and (2) the desire to interact with the same partner from neural sensitivity to social interaction in each of the two VS ROIs, for a total of four regression models. Regression models were also run for each outcome variable with neural sensitivity to social interaction in the amygdala ROI as a predictor (Supplemental information). Age, time between scan and social interaction visit, and group (AUT/TD) were included in models as covariates.

## 3 Results

### 3.1 Scanner task performance

We first examined the matched sample (*n* = 43 TD and *n* = 43 AUT). There was a significant main effect of Partner on reaction time (*F*(1, 84) = 6.67, *p* = 0.01), such that participants responded more quickly to statements sent to the peer (mean reaction time = 1.58 s) compared to the computer (mean reaction time = 1.61 s). There was no significant main effect of Group or significant interaction between Group and Partner.

For skipped statements, there were no significant main effects of Group or Partner condition, and no significant interaction between Group and Partner. The overall proportion of skipped statements was low; the participants in the AUT group skipped an average of 3.69% of statements, while participants in the TD group skipped an average of 2.62 % of statements.

### 3.2 Post-test response

**Fig. 2:**
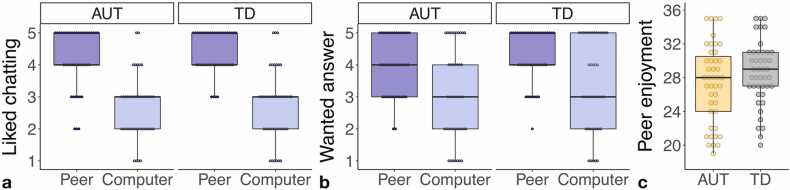
Youth in the AUT group (*n* = 43) and youth in the TD group (*n* = 43) both liked chatting with (A) and wanted to see responses from (B) the peer more than the computer. Youth in the AUT group did not differ from youth in the TD group in overall enjoyment of chatting with the scanner peer as measured across a composite of seven questions assessing peer enjoyment (C).

Participants in both the AUT and TD groups preferred the peer partner to the computer partner, with both groups reporting higher enjoyment of chatting with the peer compared to the computer partner (*p*s < 0.001; Fig. 2A) and higher desire to see the peer’s responses compared to the computer partner’s responses (*p*s < 0.001; Fig. 2B). The AUT and TD groups did not significantly differ in a composite of enjoyment of chatting with the peer partner (*t*(84) = 1.33, *p* = 0.19; Fig. 2C).

### 3.3 Whole-brain statistics

#### 3.3.1 Initiation period

There were no significant clusters of activation in the initiation period for the Peer vs. Computer contrast for either the AUT group or the TD group. Furthermore, no group differences in the response to Peer vs. Computer conditions were identified in the initiation period.

#### 3.3.2 Reply period

There were no significant group differences in the reply period for any of the contrasts.

**Fig. 3:**
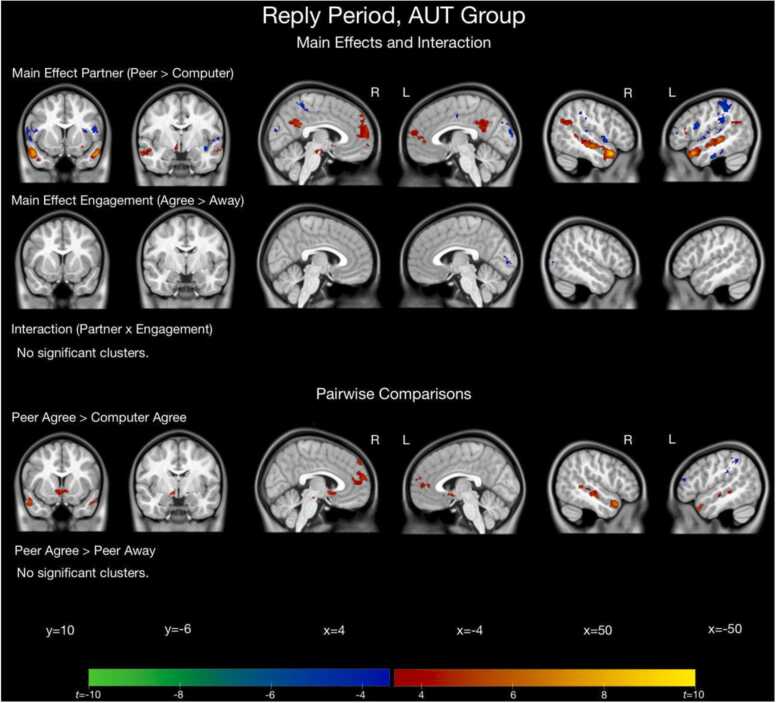
Results of whole-brain analysis for the reply period for the AUT group (*n* = 43).

**Fig. 4:**
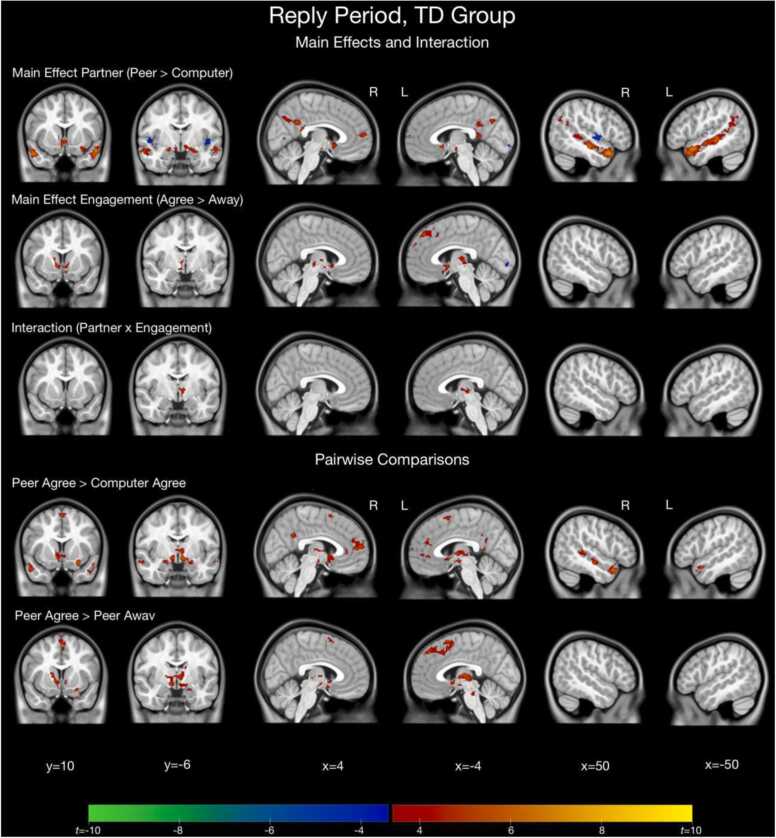
Results of whole-brain analysis for the reply period for the TD group (*n* = 43).

For the main effect of Partner, both AUT and TD groups displayed significantly greater activation to the Peer compared to Computer condition in many regions, including frontal regions, temporal regions, VS, and amygdala. For the main effect of Engagement, the TD group displayed significantly greater activation to the Agree compared to Away condition in VS and frontal regions, while the AUT group displayed significantly greater activation to the Agree compared to Away condition only in the cerebellum (see Fig. 3, Fig. 4, Supplemental Tables 3 and 4 for full details).

The pairwise contrast of Peer Agree vs. Computer Agree replies revealed similar patterns for both groups of increased activation to Peer Agree in frontal regions, temporal regions, and VS. The pairwise contrast of Peer Agree vs. Peer Away revealed no clusters of significant activation in the AUT group. In the TD group, there were significant clusters of activation for Peer Agree vs. Peer Away in VS, amygdala, and frontal regions (see Fig. 3, Fig. 4, Supplemental Tables 3 and 4 for full details of all regions activated for these contrasts for both groups).

### 3.4 ROI analyses

To specifically investigate group differences in activation to social-interactive reward within a priori defined regions, we examined interactions between Group (AUT, TD), Partner (Peer, Computer), and Engagement (Agree, Away) in the two VS ROIs.

**Fig. 5:**
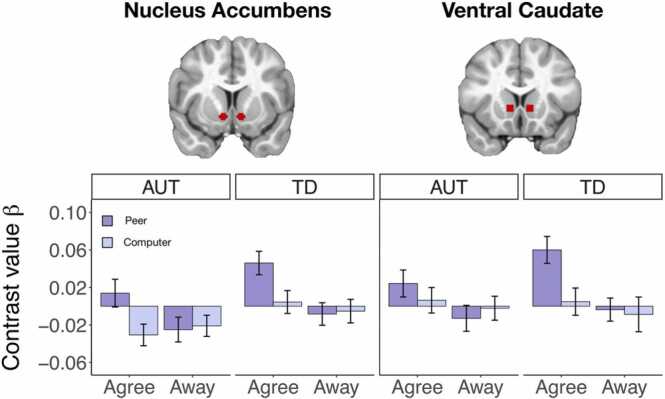
Results of ROI analyses for the nucleus accumbens and ventral caudate (*n* = 43 AUT, *n* = 43 TD). Error bars are ± standard error.

In the nucleus accumbens ROI, there was a significant main effect of Partner (*F*(1, 84) = 8.75, *p* = 0.004), a significant main effect of Engagement (*F*(1, 84) = 13.08, *p* < 0.001), and a significant interaction between Partner and Engagement (*F*(1, 84) = 13.27, *p* < 0.001; Fig. 5). Peer Agree responses showed greater activation than other reply types across groups. There was a marginal main effect of Group (*F*(1, 84) = 3.30, *p* = 0.073) such that response in the TD group was larger than response in the AUT group. However, there were no significant interactions between Group and Partner or Engagement.

In the ventral caudate ROI, there was a significant main effect of Partner (*F*(1, 84) = 4.51, *p* = 0.04), a significant main effect of Engagement (*F*(1, 84) = 18.92, *p* < 0.001) and a significant interaction between Engagement and Partner (*F*(1, 84) = 6.23, *p* = 0.01; Fig. 5), with Peer Agree responses showing greater activation than Computer Agree, Peer Away, or Computer Away responses across both groups. There was a marginally significant interaction between Group and Partner (*F*(1, 84) = 2.81, *p* = 0.098) such that response to Peer messages in the TD group was stronger than response to Peer messages in the AUT group and Computer messages in both groups. There was no significant main effect of Group or interactions between Group and any of the other factors.

For both ROIs, follow-up 2 × 2 ANOVAs to directly assess an interaction between Partner and Group within the ‘Agree’ condition did not yield significant results for the interaction term (nucleus accumbens *p* = 0.86, ventral caudate *p* = 0.09). Likewise, follow-up 2 × 2 ANOVAs to directly assess an interaction between Engagement and Group in the ‘Peer’ condition did not yield significant results for the interaction term (nucleus accumbens *p* = 0.41, ventral caudate *p* = 0.20).

Follow-up Bayesian analyses to quantify the strength of evidence for including factors of Partner, Engagement, and Group to predict neural response in the nucleus accumbens and ventral caudate ROIs revealed generally moderate to strong evidence in favor of including Partner, Engagement, and their interaction in the models. However, there was generally weak to moderate evidence *against* including Group, and interaction effects between Group and the other factors in the model, depending on the ROI (see Supplemental Tables 5 and 6).

### 3.5 Individual differences

Linear regression models were run to test age and self-reported enjoyment of chatting with the scanner peer as predictors of VS sensitivity to social interaction (i.e., the interaction term [Peer Agree – Peer Away] – [Computer Agree – Computer Away]). For these analyses, the individual differences sample (*n* = 114) was used. Group was included as a covariate in all models. Uncorrected p-values are reported. The Benjamini-Hochberg procedure was performed to account for multiple comparisons within the group of 2 VS ROIs.

#### 3.5.1 Age

Age did not significantly predict neural sensitivity to social interaction in the ventral caudate (β = − 0.01, *t*(111) = − 0.96, *p* = 0.34) or nucleus accumbens (β = − 0.01, *t*(111) = − 1.29, *p* = 0.20).

#### 3.5.2 Enjoyment of peer in scanner task

**Fig. 6:**
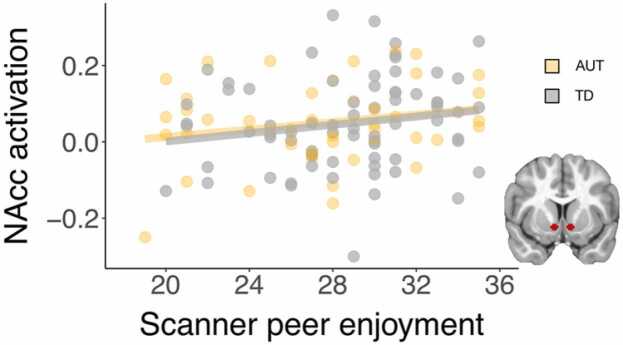
Enjoyment of the peer in the scanner task significantly predicts variation in nucleus accumbens (NAcc) sensitivity to social interaction (*n* = 114).

Enjoyment of interacting with the peer in the scanner task, as measured by the post-test peer enjoyment composite (see Supplemental information), significantly predicted neural sensitivity to social interaction in the nucleus accumbens (β = 6.69, *t*(111) = 2.00, *p* = 0.048), such that increased self-reported enjoyment of the peer predicted increased neural sensitivity to peer engaged messages controlling for other response types (Fig. 6). Correlations between nucleus accumbens sensitivity and self-reported enjoyment of the peer were similar size and direction in the both groups (TD: *r*_(69)_ = 0.17, AUT: *r*_(41)_ = 0.21). This effect was not present within the ventral caudate (β = 2.97, *t*(111) = 1.08, *p* = 0.28). The effect in the nucleus accumbens ROI did not remain statistically significant when performing the Benjamini-Hochberg procedure to account for two comparisons (both VS ROIs).

### 3.6 Longitudinal prediction of real world social-interactive behavior

**Fig. 7:**
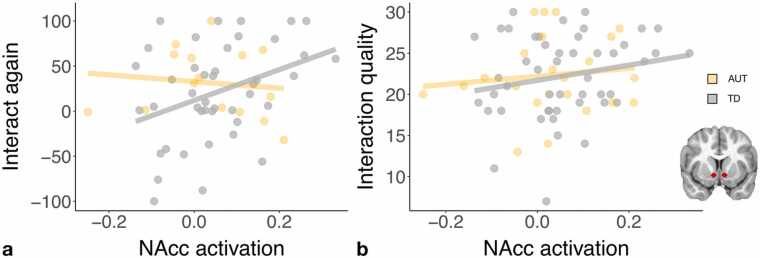
Nucleus accumbens (NAcc) sensitivity to social interaction significantly predicts desire to interact with the same partner again (A; *n* = 58), but does not significantly predict self-reported interaction quality (B; *n* = 71) during a twenty-five minute face-to-face interaction with a novel peer. Relations were marginally stronger in the TD group (grey) compared to the AUT group (orange) for desire to interact with the same partner again.

Nucleus accumbens sensitivity to social-interactive reward in the scanner significantly predicted desire to interact again with the face-to-face interaction partner at the follow-up social interaction visit (β = 112.98, *t*(53) = 2.01, *p* = 0.05; Fig. 7A). These findings held when controlling for age, time between scan and social interaction visit, and group (AUT/TD), none of which significantly predicted desire to interact with the partner again (see Supplemental Table 7). However, this effect did not remain statistically significant when performing the Benjamini-Hochberg procedure to account for multiple comparisons (2 ROIs). Nucleus accumbens sensitivity to social-interactive reward did not predict live, face-to-face interaction quality with the novel interaction partner (β = 8.11, *t*(66) = 1.52, *p* = 0.13), although the effect was in the same direction (see Fig. 7B, Supplemental Table 8).

In follow-up analyses to determine if the magnitude or direction of effects differed between the AUT and TD groups, additional linear regressions were run including the interaction between group and nucleus accumbens sensitivity to social-interactive reward (Supplemental Tables 9 and 10). There was a marginally significant interaction between nucleus accumbens sensitivity and group in predicting desire to interact with the same partner again (β = 227.31, *t*(52) = 1.80, *p* = 0.08), such that correlations between nucleus accumbens sensitivity and desire to interact with the same partner again were stronger in the TD group (TD: *r*_(41)_ = 0.37, *p* = 0.02, AUT: *r*_(13)_ = − 0.12, *p* = 0.67). This significant prediction in the TD group remained significant when performing the Benjamini-Hochberg procedure to account for multiple comparisons. Group did not significantly interact with nucleus accumbens sensitivity to predict interaction quality (β = 5.65, *t*(65) = 0.49, *p* = 0.63).

Ventral caudate sensitivity to social-interactive reward did not significantly predict interaction quality (β = − 2.09, *t*(66) = − 0.46, *p* = 0.65) or desire to interact with the same partner again (β = 58.17, *t*(53) = 1.21, *p* = 0.23).

## 4 Discussion

This study (1) investigated the neural correlates of social-interactive reward using a chat-based task in youth with autism and typical development, (2) examined factors that may account for heterogeneity in the neural response, and (3) linked this heterogeneity in social reward processing to variability in future social interaction enjoyment (i.e. desire to interact again) during a face-to-face interaction with a peer. In one of the largest studies to date to investigate social reward processing in autism, we found minimal group differences across both neural (i.e., whole-brain and ROI analyses) and behavioral (i.e., youth’s self-reported social motivation during the scanner task) social reward measures. Neural response to social-interactive reward in the VS was associated with individual differences in self-reported enjoyment of the peer partner in the fMRI task and later desire to interact with a real-life novel peer after a social interaction (i.e., enjoyment of the interaction). Together, these findings provide evidence for some commonalities in social-interactive reward in youth with and without autism and suggest a potential neural mechanism underlying variability in social-interactive enjoyment: VS response to social-interactive reward.

### 4.1 Minimal group differences in social-interactive reward processing

Analysis of both the neural response to social-interactive reward and youth’s self-reported enjoyment of the social-interactive reward task revealed minimal (i.e., non-significant) differences between the AUT and TD groups. The task activated regions in the reward and social-cognitive networks in both groups, such that whole-brain analysis did not reveal significant group differences within reward or social-cognitive regions. Additionally, neither of the a priori VS ROIs showed group differences in response to social-interactive reward. Follow-up Bayesian analyses revealed weak to moderate evidence *against* the inclusion of group or its interaction with other factors in models predicting neural response in the VS.

Instead, across both AUT and TD groups, the neural correlates of social reward (i.e., receiving a positive response from a peer) revealed involvement of brain regions implicated in reward and social-cognitive processing, including the VS, amygdala, and TPJ. These findings are consistent with previous evidence for social-interactive tasks robustly engaging brain regions in reward and social-cognitive networks in individuals with typical development (Redcay and Schilbach, 2019, Warnell et al., 2018, Alkire et al., 2018), and further extend these findings to individuals on the autism spectrum.

This absence of group differences is consistent with recent reviews and meta-analyses suggesting minimal group differences (i.e., small or no effects) in social reward processing between individuals with autism and individuals with typical development (Bottini, 2018, Clements et al., 2018). While sample selection could be a factor (i.e., those interested in participating in an experiment may be more socially motivated), these findings also align with other evidence that individuals on the autism spectrum are interested in and motivated to engage in social interaction (Jaswal and Akhtar, 2019). Interviews and self-report data from many children and adolescents on the autism spectrum indicate an interest in developing friendships and enjoyment or satisfaction with their social relationships (Calder et al., 2013, Petrina et al., 2017). Furthermore, many youth on the autism spectrum report feelings of loneliness, which suggests a desire to form strong personal connections with others (Bauminger and Kasari, 2000, Bauminger et al., 2003). These reports are in line with our findings of no significant differences between the AUT and TD groups in self-reports of liking and wanting to see the peer’s responses more than computer’s responses. Past research, combined with the current findings, present a significant challenge to the theory that autism is characterized by a “deficit” in social reward. Rather, the specific contexts in which social reward may differ between individuals with autism and typical development are likely nuanced and person dependent. For example, comorbidities with social anxiety and/or a history of peer rejection may both contribute to individual differences in social motivation (Wood and Gadow, 2010).

While our a priori hypothesis was that using more ecologically valid and interactive tasks might exacerbate group differences in social reward processing compared to prior work, our results suggest the opposite. It is possible that the ‘real-world’ context of getting to chat with a peer more naturally engaged individuals in both groups than did previous artificial paradigms, in line with evidence that children on the autism spectrum are interested in social interaction and enjoy forming new real-world relationships (Jaswal and Akhtar, 2019). Importantly, our more ecologically-valid paradigm still differed from real-world social interaction. The turn-taking of the fMRI task was highly structured, and the minimalist text-based nature of the task eliminated some of the social-cognitive, sensory, and language demands of naturalistic and unstructured face-to-face interactions that may be more challenging for some individuals on the autism spectrum. Thus, this paradigm may have reached a middle ground between higher ecological validity than non-interactive neuroimaging tasks and lower demands than real-world interactive contexts. Future research could examine neural correlates of social-interactive reward in paradigms that better approximate the social-cognitive, language, and/or sensory demands of a face-to-face, unstructured interaction to determine how these factors contribute to real-world social interaction success.

The type of reward processing examined may also have contributed to minimal group differences. Specifically, our task did not isolate the anticipation phase of reward, which may better measure the motivational component of reward. Previous rodent and human work has suggested that reward anticipation and receipt components are represented by distinct neural mechanisms (Berridge et al., 2009, Diekhof et al., 2012). Some recent work on social reward in autism has suggested that anticipation may be differentially impacted compared to receipt of reward (Keifer et al., 2021, Baumeister et al., 2020). In contrast to non-interactive paradigms that isolate anticipation and receipt by using an incentive delay structure (Rademacher et al., 2010), it is challenging to distinguish anticipation from receipt in the natural dynamics of a social interaction task. In social interactions, the reciprocal turns can encompass both enjoyment of offering information and anticipation of the partner’s response, or enjoyment of receiving the partner’s response and anticipation of the next opportunity to offer information. Therefore, the lack of group differences observed in the present study could be masking potential group differences in social reward anticipation that were not measured in our paradigm. Additionally, the paradigm used here was best designed to test differences in peer and computer feedback matched for engagement, rather than reward value per se, given the lower reported reward value of computer engagement. Future theoretical and empirical work may help to further define the constructs of anticipation and receipt in a naturalistic social interaction. Tasks that differentiate these components in ecologically valid circumstances will be able to further test hypotheses about anticipation of social reward being selectively impacted in autism.

### 4.2 Heterogeneity in neural response to social-interactive reward

Heterogeneity in social reward processing also likely contributed to the lack of group differences. Previous evidence for hyper- or hypo-activation to social reward in AUT vs. TD groups came from small samples, and therefore may reflect sampling variability in a heterogeneous population with overlapping distributions rather than true group differences. We predicted that age and social motivation might contribute to variability in social reward response (Clements et al., 2018). However, age did not significantly predict VS response. Previous investigations of age-related differences in neural response to social reward have yielded mixed results. One meta-analysis identified a large though non-significant effect of age, with increased hypo-activation to social reward in younger samples of children with autism (Clements et al., 2018). In contrast, a large study of social reward processing in autism identified effects of hypo-activation to social reward in adults but not children or adolescents with autism (Baumeister et al., 2020). The lack of age-dependent findings in our sample could reflect either a lack of consistent developmental change in VS response to social-interactive reward, or the restricted age range (7–15 years) under study here. Future longitudinal work could track changes in social reward processing in an extended age range to better understand changes in social-interactive reward processing across the lifespan.

Self-reported social enjoyment of the partner in the scanner task, however, did contribute to heterogeneity in the striatal response to social reward across both AUT and TD samples. Specifically, self-reported enjoyment of chatting with the partner in the scanner task significantly predicted nucleus accumbens response, though this finding did not hold when correcting for multiple comparisons across VS ROIs. These results emphasize the importance of considering heterogeneity in neural response as both a potential factor behind previous inconsistencies in small samples and as a source for understanding variability in processing. This consideration of neural and behavioral heterogeneity in response to social-interactive reward has important clinical implications, as it may lead to identification of subgroups, which could influence diagnosis and intervention.

There are many additional sources of heterogeneity that may contribute to reward processing variability that were not investigated in the present study, including sex and gender. Among adults with typical development, women show increased activation to social reward in the VS compared to men (Spreckelmeyer et al., 2009). In one recent study of social reward processing in autism, girls with autism showed greater nucleus accumbens activity to social reward than boys with autism (Lawrence et al., 2020). This is consistent with self-report data that indicates adolescent girls with autism report higher social motivation and better friendship quality than boys with autism (Sedgewick et al., 2016). While the present study was underpowered to directly identify sex and gender differences in autism, future work should further investigate these differences in social-interactive reward to better understand potential sex and gender differences in neural processing of social-interactive reward as another source of heterogeneity.

Another potential factor accounting for heterogeneity in social reward processing in autism could be cognitive ability. However, given the cognitive demands of the task in the current study, the youth included all had an IQ greater than 80. Therefore, the results presented may not generalize to a broader population of youth on the autism spectrum, including those with intellectual disability. Future research could develop tasks with fewer cognitive demands to investigate the neural correlates of social-interactive reward processing in individuals with autism and intellectual disability.

The degree of autistic traits may also contribute to heterogeneity in reward processing in both the AUT and TD samples. Although the current study did not collect a continuous measure of autistic traits for all participants, future work could make use of instruments that measure autistic traits continuously in populations with and without autism to best evaluate levels of autistic traits as a potential source of heterogeneity.

### 4.3 Neural response to social-interactive reward predicts real-world enjoyment of face-to-face interaction with a novel peer

In the present study, heterogeneity in VS response not only correlated with enjoyment of chatting with the peer in that scanner task, but predicted enjoyment of a face-to-face interaction, i.e. the desire to interact again, with a novel peer several months or even years later. Therefore, VS sensitivity to social reward as indexed in our paradigm may capture something more general about social enjoyment rather than being specific to a particular task or interaction. Previous work has demonstrated that adolescents’ neural response to social reward predicts their positive experiences with groups of familiar peers (Alarcón et al., 2020) and their average self-reported feelings of closeness in social interactions as assessed with ecological momentary assessment (Flores et al., 2018). The present analyses provide novel evidence that neural response to social-interactive reward predicts an aspect of initial interaction enjoyment, the desire to interact again with a novel peer.

The desire to interact again with the novel peer partner significantly correlated with VS response to social-interactive reward in the TD group, but the correlation between the two variables in the AUT group was near zero. Given the small sample size in the AUT group relative to the TD group, this finding of a relation in typical development but not autism should be taken as preliminary. Future research could confirm these real-world relations, which are in line with previous theories about the anticipation component of reward being specifically impacted in autism. That is, VS sensitivity to the social reward may influence enjoyment of an interaction, but this relation may be altered in autism when predicting motivation to engage with a peer again.

Overall, this study underscores commonalities in social-interactive reward processing in autism and typical development and the importance of considering heterogeneity in social reward responsivity in autism and typical development. We found that heterogeneity in VS response to social-interactive reward had implications for predicting future enjoyment of initial social interactions, particularly in youth with typical development. Therefore, we highlight the importance of ecologically valid approaches to better understand social interaction challenges in autism.

## CRediT authorship contribution statement

K.A.M, L.A.K., K.R.W., D.A., and E.R. designed the experiments. K.A.M., L.A.K., K.R.W, D.M., D.A., and H.Y collected data. L.A.K., H.Y., and A.T. performed the clinical characterization. K.A.M. and J.M. analyzed the data. D.M. contributed analytic code. E.R. supervised the data collection and analysis. K.A.M. drafted the initial manuscript. All authors revised the manuscript and approved the final version for submission.

## Funding

Research reported in this publication was supported by the National Institute of Mental Health of the 10.13039/100000002National Institutes of Health under Award nos. R01MH107441 (PI: Redcay) and F31MH127781 (PI: McNaughton). The content is solely the responsibility of the authors and does not necessarily represent the official views of the National Institutes of Health. D.A. was involved in this study and other research supported by R01MH107441 in her previous role as a doctoral student and postdoctoral associate prior to her employment with the 10.13039/100000026National Institute on Drug Abuse.

## Declaration of Competing Interest

The authors declare that they have no known competing financial interests or personal relationships that could have appeared to influence the work reported in this paper.

## Data Availability

The data that support the findings described in this manuscript are available from the corresponding author upon reasonable request.
