# Supplementary material for: Social-interactive reward elicits similar neural response in autism and typical development and predicts future social experiences
Source: Dev Cogn Neurosci. 2023 Jan 6;59:101197. doi: 10.1016/j.dcn.2023.101197 (PMC9852551; doi:10.1016/j.dcn.2023.101197)
Supplement: Supplementary file 1 — Supplementary material [file mmc1.docx]

**Supplemental Information**

Additional Demographic Information 1

Additional Task Information 3

Whole-Brain Analyses 4

Bayesian Repeated Measures ANOVAs 15

Regression Models Predicting Social Interaction Outcomes from Nucleus Accumbens Response 16

Amygdala Results 18

fMRIPrep Preprocessing 23

Post-Test 28

Social Interaction Slider Question 33

# Additional Demographic Information

*Supplemental Table 1. Participant Race, Ethnicity, and Household Income.*

| **Race** | **Percentage of Sample (*n*=114)** |
| --- | --- |
| Asian | 2 |
| Black or African American | 11 |
| White | 67 |
| More than One Race | 16 |
| Missing or Did Not Wish to Report | 4 |
| **Ethnicity** | |
| Hispanic/Latino | 7 |
| Not Hispanic/Latino | 89 |
| Missing or Did Not Wish to Report | 4 |
| **Household Income** | |
| $75,000 or more | 83 |
| $35,000-$75,000 | 11 |
| Less than $35,000 | 1 |
| Missing or Did Not Wish to Report | 5 |

*Supplemental Table 2. Means and Standard Deviations for Behavioral Measures by Group.*

| Measure | TD Group  Mean (SD) | AUT Group  Mean (SD) |
| --- | --- | --- |
| Reaction Time to Peer Messages (seconds)^1^ | 1.57 (0.28) | 1.58 (0.24) |
| Reaction Time to Computer Messages (seconds)^1^ | 1.59 (0.29) | 1.64 (0.28) |
| Percent of Skipped Responses^1^ | 2.62 (3.47) | 3.69 (5.35) |
| Post-test Peer Enjoyment Composite^2^ | 28.6 (3.85) | 27.3 (4.64) |
| Interaction Quality^3^ | 22.2 (5.06) | 22.3 (4.73) |
| Desire to Interact Again^4^ | 21.0 (53.2) | 31.3 (38.2) |

Note: Sample size varies by measure. 1=matched sample (*n*=43 TD, *n*=43 AUT), 2=full sample (*n*=71 TD, *n*=43 AUT), 3=follow-up social interaction sample that completed interaction quality questionnaire (*n*=48 TD, *n*=23 AUT), 4=follow-up social interaction sample that completed desire to interact again question (*n*=43 TD, *n*=15 AUT).

# Additional Task Information


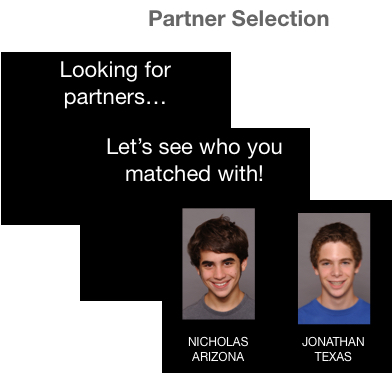


*Supplemental Figure 1.* After learning about the chat game, children chose their chat partner from two potential partners. Potential partners were age and gender-matched to the child. Images of potential partners were chosen from the NIMH-ChEFs set (Egger et al., 2011; direct gaze, smiling only), Getty Images, or Google Images search. Participants also had their photo taken and were told their photo would be sent to their chat partner to enhance believability of the live illusion.

# Whole-Brain Analyses

*Supplemental Table 3. Whole-brain analysis of regions activated during the social-interactive reward task in the AUT group.*

Initiation Period

Effect of Peer Initiation vs. Computer Initiation

None.

Reply Period

Main Effect of Partner

Peer > Computer

|  | | | | MNI Coordinates | | |
| --- | --- | --- | --- | --- | --- | --- |
| Region | Side | Peak *t* | Cluster *k* | x | y | z |
| Temporal Pole (extending into  Middle Temporal Gyrus  Frontal Orbital Cortex  Insula) | R | 9.18 | 2206 | 50 | 14 | -24 |
|  | R |  |  |  |  |  |
|  | R |  |  |  |  |  |
|  | R |  |  |  |  |  |
| Temporal Pole (extending into  Middle Temporal Gyrus  Frontal Orbital Cortex  Inferior Frontal Gyrus (p. triangularis)  Insula) | L | 8.29 | 1964 | -50 | 12 | -26 |
|  | L |  |  |  |  |  |
|  | L |  |  |  |  |  |
|  | L |  |  |  |  |  |
|  | L |  |  |  |  |  |
| Frontal Pole (extending into  Dorsomedial Prefrontal Cortex (dmPFC)  Ventromedial Prefrontal Cortex (vmPFC)) | R | 6.55 | 1259 | 8 | 54 | 32 |
|  | L/R |  |  |  |  |  |
|  | L/R |  |  |  |  |  |
| Posterior Cingulate Cortex (extending bilaterally) | R | 5.95 | 888 | 6 | -48 | 26 |
| Angular Gyrus (extending into  Temporoparietal Junction (TPJ)) | R | 5.58 | 412 | 54 | -60 | 24 |
|  | R |  |  |  |  |  |
| Hypothalamus (extending into  Caudate  Amygdala) | R | 5.67 | 236 | 8 | -4 | -8 |
|  | R |  |  |  |  |  |
|  | R |  |  |  |  |  |
| Cerebellum | L | 5.64 | 234 | -24 | -76 | -32 |
| Lateral Occipital Cortex (extending into  Angular Gyrus) | L | 5.28 | 191 | -46 | -64 | 26 |
|  | L |  |  |  |  |  |
| Frontal Pole | L | 5.27 | 153 | -10 | 40 | 46 |

Computer > Peer

|  |  |  |  |  | MNI Coordinates | | |
| --- | --- | --- | --- | --- | --- | --- | --- |
| Region | | Side | Peak *t* | Cluster *k* | x | y | z |
| Supramarginal gyrus (extending into  Planum Temporale) | | L | 7.2 | 1462 | -62 | -34 | 42 |
|  |  | L |  |  |  |  |  |
| Supramarginal Gyrus (extending into  Planum Temporale) | | R | 5.57 | 1064 | 62 | -34 | 46 |
|  |  | R |  |  |  |  |  |
| Occipital Pole (extending into  Cuneus) | | R | 8.65 | 1003 | 14 | -90 | 2 |
|  |  | R |  |  |  |  |  |
| Precentral Gyrus (extending into  Insula  Inferior Frontal Gyrus (p. opercularis)) | | L | 5.75 | 776 | -50 | 4 | 20 |
|  |  | L |  |  |  |  |  |
|  |  | L |  |  |  |  |  |
| Precentral Gyrus (extending into  Precuneus) | | R | 5.48 | 435 | 18 | -38 | 44 |
|  |  | R |  |  |  |  |  |
| Cingulate (extending into  Precuneus  Precentral Gyrus) | | L | 5.28 | 413 | -14 | -34 | 38 |
|  |  | L |  |  |  |  |  |
|  |  | L |  |  |  |  |  |
| Fusiform Cortex | | L | 5.08 | 373 | -26 | -48 | -16 |
| Frontal Pole | | L | 5.74 | 337 | -46 | 44 | 20 |
| Precentral Gyrus (extending into  Postcentral Gyrus) | | L | 5.59 | 320 | -18 | -28 | 64 |
|  |  | L |  |  |  |  |  |
| Inferior Temporal Gyrus | | L | 7.3 | 295 | -46 | -18 | -30 |
| Lingual Gyrus (extending into  Fusiform Cortex) | | R | 6.16 | 291 | 26 | -46 | -8 |
|  |  | R |  |  |  |  |  |
| Occipital Pole | | L | 5.93 | 238 | -12 | -96 | 0 |
| Occipital Pole | | L | 5.99 | 232 | -10 | -96 | 28 |
| Precentral Gyrus | | R | 4.97 | 189 | 26 | -24 | 54 |

Main Effect of Engagement

Agree > Away

|  |  |  |  |  | MNI Coordinates | | |
| --- | --- | --- | --- | --- | --- | --- | --- |
| Region | | Side | Peak *t* | Cluster *k* | x | y | z |
| Cerebellum | | R | 5.32 | 171 | 36 | -68 | -38 |

Away > Agree

|  |  |  |  |  | MNI Coordinates | | |
| --- | --- | --- | --- | --- | --- | --- | --- |
| Region | | Side | Peak t | Cluster k | x | y | z |
| Occipital Pole (extending into  Lingual Gyrus) | | L | 7.03 | 771 | -12 | -94 | 4 |
|  |  | L |  |  |  |  |  |
| Occipital Pole | | R | 5.89 | 489 | 26 | -96 | 22 |
| Lateral Occipital Cortex (extending into  Fusiform Cortex) | | R | 5.21 | 345 | 38 | -74 | -10 |
|  |  | R |  |  |  |  |  |
| Lateral Occipital Cortex (extending into  Precuneus) | | R | 4.8 | 148 | 20 | -72 | 38 |
|  |  | R |  |  |  |  |  |

Interaction Effect (Partner x Engagement)

None.

Peer Agree vs. Computer Agree

Peer Agree > Computer Agree

|  |  |  |  |  | MNI Coordinates | | |
| --- | --- | --- | --- | --- | --- | --- | --- |
| Region | | Side | Peak *t* | Cluster *k* | x | y | z |
| Frontal Pole (extending into  dmPFC  Anterior Cingulate Cortex) | | R | 6.04 | 964 | 8 | 54 | 32 |
|  |  | L/R |  |  |  |  |  |
|  |  | L/R |  |  |  |  |  |
| Middle Temporal Gyrus | | R | 5.9 | 594 | 48 | -44 | 4 |
| Pallidum (extending bilaterally into  Parahippocampal Gyrus  Nucleus Accumbens  Anterior Cingulate Cortex) | | R | 6 | 461 | 10 | 0 | -10 |
|  |  | L/R |  |  |  |  |  |
|  |  | L/R |  |  |  |  |  |
|  |  | L/R |  |  |  |  |  |
| Temporal Pole (extending into  Frontal Orbital Cortex  Insula) | | R | 6.86 | 396 | 52 | 14 | -26 |
|  |  | R |  |  |  |  |  |
|  |  | R |  |  |  |  |  |
| Middle Temporal Gyrus | | L | 5.31 | 225 | -54 | -34 | -2 |
| Temporal Pole | | L | 5.83 | 219 | -50 | 14 | -32 |

Computer Agree > Peer Agree

|  |  |  |  |  | MNI Coordinates | | |
| --- | --- | --- | --- | --- | --- | --- | --- |
| Region | | Side | Peak *t* | Cluster *k* | x | y | z |
| Supramarginal Gyrus | | L | 5.38 | 296 | -52 | -46 | 46 |
| Fusiform Cortex | | R | 6.17 | 172 | 30 | -52 | -12 |
| Frontal Pole | | L | 5.65 | 171 | -46 | 44 | 14 |

Peer Agree vs. Peer Away

Peer Agree > Peer Away

None.

Peer Away > Peer Agree

None.

Peer Away vs. Computer Away

Peer Away > Computer Away

|  |  |  |  |  | MNI Coordinates | | |
| --- | --- | --- | --- | --- | --- | --- | --- |
| Region | | Side | Peak *t* | Cluster *k* | x | y | z |
| Temporal Pole (extending into  Middle Temporal Gyrus) | | R | 7.32 | 1363 | 50 | 14 | -26 |
|  |  | R |  |  |  |  |  |
| Temporal Pole (extending into  Middle Temporal Gyrus) | | L | 7.3 | 1098 | -54 | 8 | -20 |
|  |  | L |  |  |  |  |  |
| Precuneus (extending bilaterally into  Cingulate Cortex) | | L | 5.58 | 544 | -8 | -62 | 32 |
|  |  | L/R |  |  |  |  |  |
| Angular Gyrus (extending into  TPJ) | | L | 5.82 | 312 | -44 | -62 | 26 |
|  |  | L |  |  |  |  |  |
| Angular Gyrus (extending into  TPJ) | | R | 5.21 | 174 | 54 | -60 | 22 |
|  |  | R |  |  |  |  |  |

Computer Away > Peer Away

|  |  |  |  |  | MNI Coordinates | | |
| --- | --- | --- | --- | --- | --- | --- | --- |
| Region | | Side | Peak *t* | Cluster *k* | x | y | z |
| Supramarginal Gyrus (extending into  Planum Temporale) | | L | 6.4 | 1060 | -60 | -42 | 46 |
|  |  | L |  |  |  |  |  |
| Occipital Pole (extending into  Cuneus) | | R | 6.94 | 1035 | 12 | -90 | 2 |
|  |  | L/R |  |  |  |  |  |
| Precentral Gyrus (extending into  Inferior Frontal Gyrus (p. opercularis)  Planum Temporale  Supramarginal Gyrus) | | R | 6.22 | 823 | 60 | 2 | 8 |
|  |  | R |  |  |  |  |  |
|  |  | R |  |  |  |  |  |
|  |  | R |  |  |  |  |  |
| Postcentral Gyrus (extending into  Superior Frontal Gyrus) | | R | 4.73 | 424 | 30 | -28 | 64 |
|  |  | R |  |  |  |  |  |
| Postcentral Gyrus | | L | 5.74 | 402 | -20 | -38 | 66 |
| Lingual Gyrus (extending into  Occipital Pole) | | L | 5.75 | 340 | -10 | -88 | -6 |
|  |  | L |  |  |  |  |  |
| Insula | | L | 6.56 | 274 | -38 | -18 | -6 |
| Cingulate Cortex (extending into  Precentral Gyrus) | | L | 5.24 | 185 | -10 | -20 | 42 |
|  |  | L |  |  |  |  |  |
| Fusiform Cortex | | L | 6.54 | 165 | -44 | -34 | -22 |
| Precentral Gyrus | | R | 4.58 | 154 | 12 | -36 | 46 |

Computer Agree vs. Computer Away

Computer Agree > Computer Away

None.

Computer Away > Computer Agree

|  |  |  |  |  | MNI Coordinates | | |
| --- | --- | --- | --- | --- | --- | --- | --- |
| Region | | Side | Peak *t* | Cluster *k* | x | y | z |
| Occipital Pole (extending into  Lingual Gyrus) | | L | 6.68 | 1051 | -12 | -94 | 4 |
|  |  | L |  |  |  |  |  |
| Fusiform Cortex (extending into  Occipital Pole) | | R | 5.1 | 426 | 28 | -76 | -6 |
|  |  | R |  |  |  |  |  |
| Precentral Gyrus | | L | 4.76 | 144 | -28 | -8 | 66 |

*Supplemental Table 4. Whole-brain analysis of regions activated during the social-interactive reward task in the TD group.*

Initiation Period

Effect of Peer Initiation vs. Computer Initiation

None.

Reply Period

Main Effect of Partner

Peer > Computer

|  |  |  |  |  | MNI Coordinates | | |
| --- | --- | --- | --- | --- | --- | --- | --- |
| Region | | Side | Peak *t* | Cluster *k* | x | y | z |
| Temporal Pole (extending into  Middle Temporal Gyrus  Frontal Orbital Cortex  TPJ  Insula) | | L | 8.17 | 1974 | -52 | 10 | -20 |
|  |  | L |  |  |  |  |  |
|  |  | L |  |  |  |  |  |
|  |  | L |  |  |  |  |  |
|  |  | L |  |  |  |  |  |
| Temporal Pole (extending into  Middle Temporal Gyrus) | | R | 7.36 | 1278 | 48 | 14 | -26 |
|  |  | R |  |  |  |  |  |
| Posterior Cingulate (extending bilaterally into  Precuneus) | | R | 6.54 | 1168 | 8 | -50 | 30 |
|  |  | L/R |  |  |  |  |  |
| Nucleus Accumbens (extending bilaterally into  Caudate  Subgenual Anterior Cingulate Cortex) | | R | 5.16 | 261 | 4 | 12 | -8 |
|  |  | R |  |  |  |  |  |
|  |  | L/R |  |  |  |  |  |
| Frontal Pole (extending bilaterally) | | R | 5.24 | 232 | 4 | 60 | 10 |
| Lateral Occipital Cortex | | R | 5.27 | 212 | 60 | -62 | 26 |
| Amygdala | | L | 5.28 | 201 | -20 | -10 | -12 |
| Amygdala | | R | 5.34 | 150 | 22 | -6 | -14 |

Computer > Peer

|  |  |  |  |  | MNI Coordinates | | |
| --- | --- | --- | --- | --- | --- | --- | --- |
| Region | | Side | Peak *t* | Cluster *k* | x | y | z |
| Occipital Pole | | R | 7.68 | 423 | 16 | -90 | 0 |
| Superior Temporal Gyrus (extending into  Insula) | | R | 5.51 | 213 | 50 | -4 | 0 |
|  |  | R |  |  |  |  |  |
| Insula | | L | 5 | 205 | -40 | -18 | -6 |
| Occipital Pole | | L | 6.04 | 191 | -10 | -94 | 0 |

Main Effect of Engagement

Agree > Away

|  |  |  |  |  | MNI Coordinates | | |
| --- | --- | --- | --- | --- | --- | --- | --- |
| Region | | Side | Peak *t* | Cluster *k* | x | y | z |
| Superior Frontal Gyrus (extending into  dmPFC  Paracingulate Gyrus) | | L | 5.58 | 642 | -4 | 44 | 46 |
|  |  | L |  |  |  |  |  |
|  |  | L |  |  |  |  |  |
| Orbitofrontal Cortex (extending into  Caudate  Nucleus Accumbens) | | L | 6.14 | 408 | -6 | 14 | -18 |
|  |  | L/R |  |  |  |  |  |
|  |  | L/R |  |  |  |  |  |
| Inferior Frontal Gyrus (p. triangularis) | | L | 5.4 | 179 | -40 | 36 | 0 |
| Middle Temporal Gyrus | | L | 5.5 | 168 | -66 | -36 | -16 |
| Thalamus (extending bilaterally) | | L | 4.86 | 147 | -4 | -22 | 4 |

Away > Agree

|  |  |  |  |  | MNI Coordinates | | |
| --- | --- | --- | --- | --- | --- | --- | --- |
| Region | | Side | Peak *t* | Cluster *k* | x | y | z |
| Occipital Pole | | L | 6.63 | 289 | -12 | -96 | -2 |

Interaction Effect (Partner x Engagement)

|  |  |  |  |  | MNI Coordinates | | |
| --- | --- | --- | --- | --- | --- | --- | --- |
| Region | | Side | Peak *t* | Cluster *k* | x | y | z |
| Thalamus (extending into  Pallidum) | | L | 4.76 | 161 | -8 | -4 | 4 |
|  |  | L |  |  |  |  |  |

Peer Agree vs. Computer Agree

Peer Agree > Computer Agree

|  |  |  |  |  | MNI Coordinates | | |
| --- | --- | --- | --- | --- | --- | --- | --- |
| Region | | Side | Peak *t* | Cluster *k* | x | y | z |
| Frontal Orbital Cortex (extending into  Amygdala  Caudate  Nucleus Accumbens  Anterior Cingulate  Insula  Substantia Nigra  Temporal Pole  Pallidum  Thalamus) | | L  L/R  L/R  L/R  L/R  L  L/R  L  L/R  L/R | 7.42 | 1758 | -28 | 14 | -20 |
| Temporal Pole (extending into  Middle Temporal Gyrus) | | R | 6.58 | 692 | 48 | 14 | -26 |
|  |  | R |  |  |  |  |  |
| Frontal Pole (extending bilaterally into  Medial Prefrontal Cortex  Anterior Cingulate Cortex) | | R | 5.88 | 623 | 6 | 52 | 8 |
|  |  | L/R |  |  |  |  |  |
|  |  | L/R |  |  |  |  |  |
| Cingulate Cortex | | R | 5.33 | 299 | 2 | -52 | 30 |
| Frontal Orbital Cortex (extending into  Insula) | | R | 6.9 | 174 | 28 | 18 | -14 |
|  |  | R |  |  |  |  |  |
| Superior Frontal Gyrus | | L/R | 5.61 | 150 | 0 | 10 | 60 |

Computer Agree > Peer Agree

None.

Peer Agree vs. Peer Away

Peer Agree > Peer Away

|  |  |  |  |  | MNI Coordinates | | |
| --- | --- | --- | --- | --- | --- | --- | --- |
| Region | | Side | Peak *t* | Cluster *k* | x | y | z |
| Thalamus (extending bilaterally into  Substantia Nigra  Caudate  Pallidum  Amygdala  Nucleus Accumbens) | | L | 6.36 | 1389 | -2 | -14 | 6 |
|  |  | L/R |  |  |  |  |  |
|  |  | L/R |  |  |  |  |  |
|  |  | L/R |  |  |  |  |  |
|  |  | L |  |  |  |  |  |
|  |  | L/R |  |  |  |  |  |
| Superior Frontal Gyrus (extending bilaterally into  dmPFC) | | L | 5.94 | 745 | -2 | 12 | 62 |
|  |  | L |  |  |  |  |  |
| Frontal Orbital Cortex | | L | 6.24 | 356 | -24 | 16 | -22 |

Peer Away > Peer Agree

None.

Peer Away vs. Computer Away

Peer Away > Computer Away

|  |  |  |  |  | MNI Coordinates | | |
| --- | --- | --- | --- | --- | --- | --- | --- |
| Region | | Side | Peak *t* | Cluster *k* | x | y | z |
| Middle Temporal Gyrus (extending into  Temporal Pole  TPJ) | | L | 6.56 | 1743 | -54 | 2 | -22 |
|  |  | L |  |  |  |  |  |
|  |  | L |  |  |  |  |  |
| Middle Temporal Gyrus (extending into  Temporal Pole) | | R | 6.27 | 627 | 56 | 0 | -20 |
|  |  | R |  |  |  |  |  |
| Posterior Cingulate (extending bilaterally into  Precuneus) | | L | 5.42 | 319 | -6 | -46 | 26 |
|  |  | L |  |  |  |  |  |
| Lateral Occipital Cortex (extending into  TPJ) | | R | 5.38 | 171 | 60 | -64 | 28 |
|  |  | R |  |  |  |  |  |

Computer Away > Peer Away

|  |  |  |  |  | MNI Coordinates | | |
| --- | --- | --- | --- | --- | --- | --- | --- |
| Region | | Side | Peak *t* | Cluster *k* | x | y | z |
| Insula | | R | 5.76 | 490 | 50 | -6 | 6 |
| Insula | | L | 5.44 | 380 | -40 | -18 | -4 |
| Occipital Pole | | R | 6.65 | 325 | 14 | -90 | -2 |
| Occipital Pole | | L | 5.2 | 159 | -12 | -92 | 2 |

Computer Agree vs. Computer Away

Computer Agree > Computer Away

None.

Computer Away > Computer Agree

|  |  |  |  |  | MNI Coordinates | | |
| --- | --- | --- | --- | --- | --- | --- | --- |
| Region | | Side | Peak *t* | Cluster *k* | x | y | z |
| Occipital Pole | | L | 5.8 | 211 | -10 | -92 | -2 |


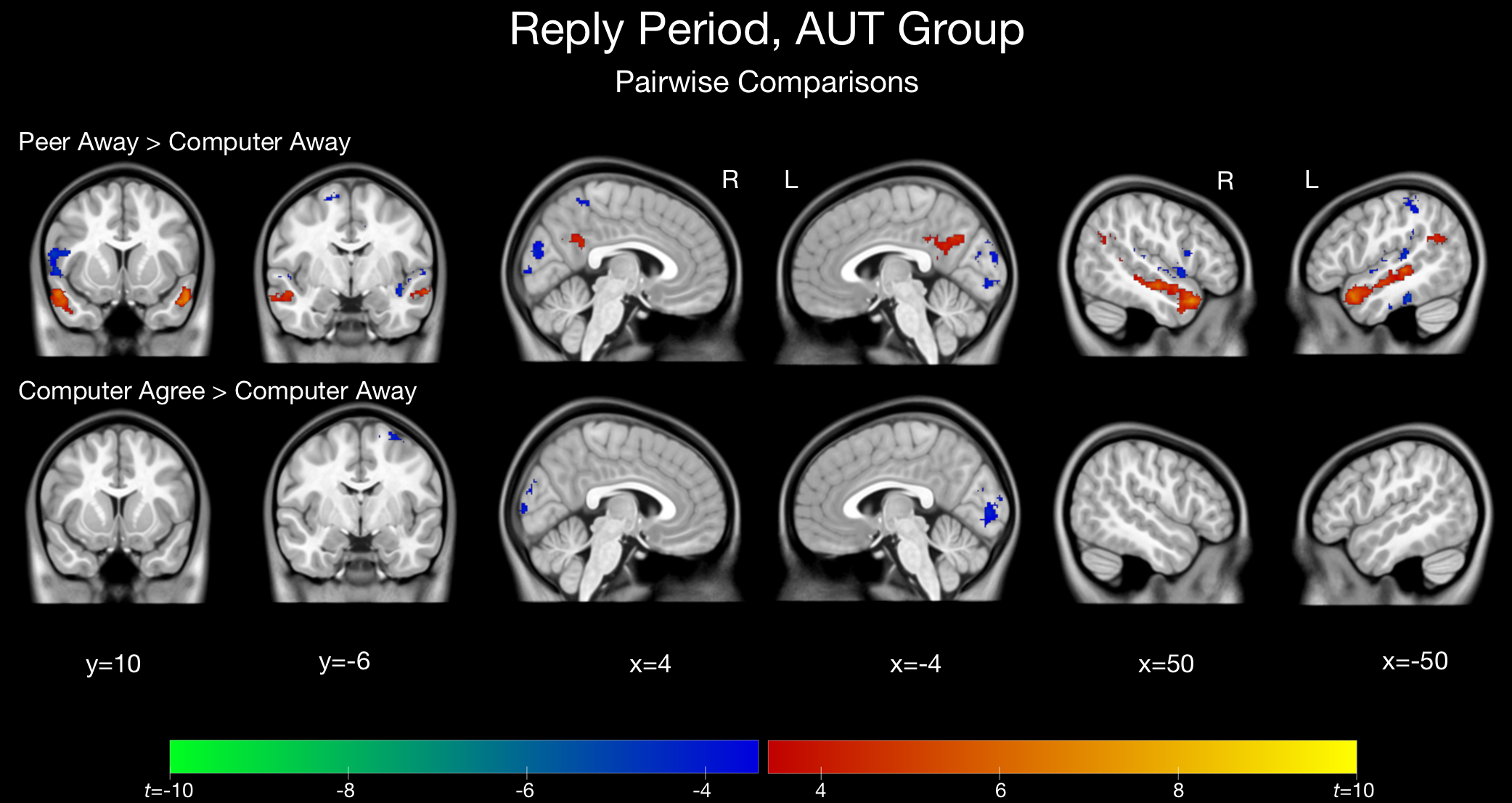


*Supplemental Figure 2.* Results of whole-brain analysis for two pairwise contrasts for the reply period for the AUT group (*n*=43).


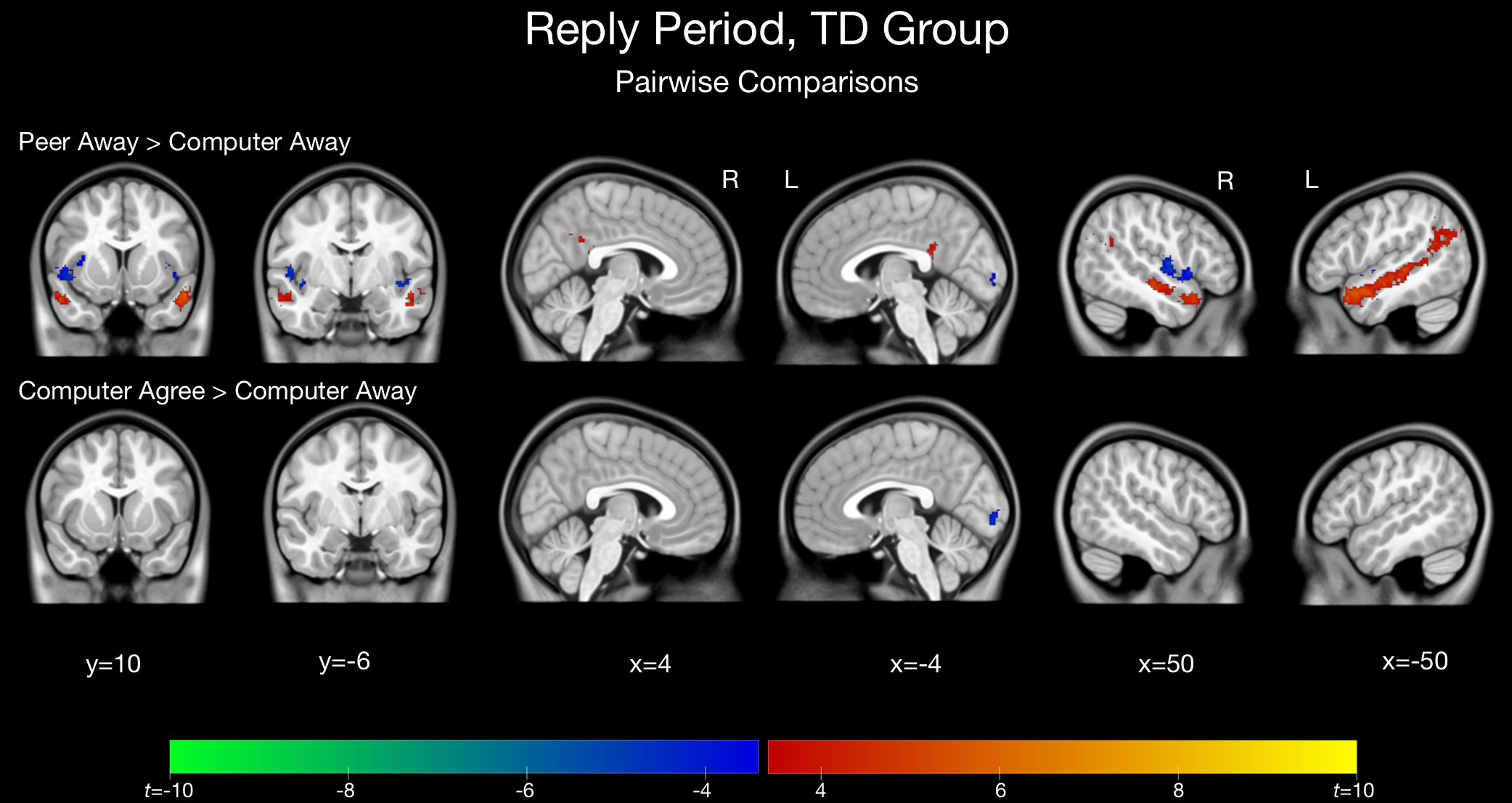


*Supplemental Figure 3.* Results of whole-brain analysis for two pairwise contrasts for the reply period for the TD group (*n*=43).

# Bayesian Repeated Measures ANOVAs

Bayesian repeated measures ANOVAs were conducted in JASP v0.14.1 to quantify evidence for inclusion or exclusion of effects, comparing among matched models.

*Supplemental Table 5. Bayes factors for inclusion of the effects of partner, engagement, and group in nucleus accumbens response.*

| Effects | BF_incl_ | BF_excl_ |
| --- | --- | --- |
| Partner | 8.132 | 0.123 |
| Engagement | 38.32 | 0.026 |
| Group | 1.109 | 0.901 |
| Partner * Engagement | 62.11 | 0.016 |
| Partner * Group | 0.174 | 5.747 |
| Engagement * Group | 0.355 | 2.817 |
| Partner * Engagement * Group | 0.211 | 4.739 |

*Supplemental Table 6. Bayes factors for inclusion of the effects of partner, engagement, and group in ventral caudate response.*

| Effects | BF_incl_ | BF_excl_ |
| --- | --- | --- |
| Partner | 1.361 | .735 |
| Engagement | 199.8 | 0.005 |
| Group | 0.244 | 4.098 |
| Partner * Engagement | 3.060 | 0.327 |
| Partner * Group | 0.670 | 1.493 |
| Engagement * Group | 0.279 | 3.584 |
| Partner * Engagement * Group | 0.349 | 2.87 |

# Regression Models Predicting Social Interaction Outcomes from Nucleus Accumbens Response

Two regression models were run to predict interaction quality and desire to interact again from nucleus accumbens sensitivity to social-interactive reward.

*Supplemental Table 7. Regression table for analysis predicting desire to interact with same partner from nucleus accumbens sensitivity to social-interactive reward.*

|  | Estimate | Standard Error | *t* | *p* |
| --- | --- | --- | --- | --- |
| Intercept | 32.91 | 51.98 | 0.63 | 0.53 |
| Nucleus accumbens activation | 112.98 | 56.33 | 2.01 | 0.05 |
| Group | -7.55 | 16.69 | -0.45 | 0.65 |
| Age at follow-up visit | 0.12 | 3.97 | 0.03 | 0.98 |
| Time between scan and follow-up visit | -5.62 | 7.05 | -0.80 | 0.43 |

*Supplemental Table 8. Regression table for analysis predicting interaction quality from nucleus accumbens sensitivity to social-interactive reward.*

|  | Estimate | Standard Error | *t* | *p* |
| --- | --- | --- | --- | --- |
| Intercept | 22.03 | 4.71 | 4.68 | <0.001 |
| Nucleus accumbens activation | 8.11 | 5.33 | 1.52 | 0.13 |
| Group | 0.15 | 1.42 | 0.11 | 0.92 |
| Age at follow-up visit | 0.07 | 0.36 | 0.20 | 0.84 |
| Time between scan and follow-up visit | -0.63 | 0.66 | -0.95 | 0.34 |

Follow-up models were run including the interaction between group (AUT/TD) and nucleus accumbens sensitivity to social-interactive reward to test for differences between the groups in the strength of the relation between nucleus accumbens sensitivity to social reward and social interaction enjoyment.

*Supplemental Table 9. Regression table for analysis predicting desire to interact with same partner from nucleus accumbens sensitivity to social-interactive reward including interaction with group.*

|  | Estimate | Standard Error | *t* | *p* |
| --- | --- | --- | --- | --- |
| Intercept | 17.35 | 51.64 | 0.34 | 0.74 |
| Nucleus accumbens activation | -48.71 | 105.44 | -0.46 | 0.65 |
| Group | -14.21 | 16.76 | -0.85 | 0.40 |
| Age at follow-up visit | 1.92 | 4.02 | 0.48 | 0.64 |
| Time between scan and follow-up visit | -7.52 | 6.98 | -1.08 | 0.29 |
| Nucleus accumbens * Group | 227.31 | 126.32 | 1.80 | 0.08 |

*Supplemental Table 10. Regression table for analysis predicting interaction quality from nucleus accumbens sensitivity to social-interactive reward including interaction with group.*

|  | Estimate | Standard Error | *t* | *p* |
| --- | --- | --- | --- | --- |
| Intercept | 21.68 | 4.79 | 4.53 | <0.001 |
| Nucleus accumbens activation | 4.37 | 9.36 | 0.47 | 0.64 |
| Group | -0.01 | 1.46 | -0.01 | 0.99 |
| Age at follow-up visit | 0.11 | 0.37 | 0.31 | 0.76 |
| Time between scan and follow-up visit | -0.68 | 0.68 | -1.01 | 0.32 |
| Nucleus accumbens * Group | 5.65 | 11.62 | 0.49 | 0.63 |

# Amygdala Results

The amygdala has previously been implicated in social reward processing differences in autism as well as social-interactive reward processing in children with typical development. Therefore, we also used an anatomically defined bilateral amygdala ROI to examine group differences in amygdala response to social-interactive reward, to test whether age and self-reported enjoyment of the peer in the scanner task predicted heterogeneity in amygdala response, and to test whether amygdala response significantly predicted later face-to-face interaction enjoyment with a peer partner.

We examined interactions between Group (AUT, TD), Partner (Peer, Computer), and Engagement (Agree, Away) in the amygdala ROI during the Reply period. In the amygdala ROI, there was a significant main effect of Partner (*F*(1, 84)=11.00, *p*=0.001), a significant main effect of Engagement (*F*(1, 84)=23.21, *p*<0.001), and a significant interaction between Partner and Engagement (*F*(1, 84)=8.28, *p*=0.005). There was no significant main effect of Group, and there were no interactions between Group and either Partner or Engagement (Supplemental Figure 4).

*Supplemental Figure 4.* Results of ROI analyses for the amygdala (*n*=43 AUT, *n*=43 TD). Error bars are +/- standard error.

Bayesian analyses revealed moderate to strong evidence in favor of including Partner, Engagement, and their interaction to predict neural response in the ventral caudate (Supplemental Table 11). However, there was moderate evidence against the inclusion of Group and its interactions with other factors in the model.

*Supplemental Table 11. Bayes factors for inclusion of the effects of partner, engagement, and group in amygdala response.*

| Effects | BF_incl_ | BF_excl_ |
| --- | --- | --- |
| Partner | 75.62 | 0.013 |
| Engagement | 801.2 | 0.001 |
| Group | 0.242 | 4.13 |
| Partner * Engagement | 5.446 | 0.184 |
| Partner * Group | 0.288 | 3.47 |
| Engagement * Group | 0.166 | 6.02 |
| Partner * Engagement * Group | 0.236 | 4.24 |

Two regression models were constructed to predict neural sensitivity to social reward in the amygdala: one predicting amygdala response from age and the other predicting amygdala response from self-reported experience of social reward during the fMRI task. Neural sensitivity to social-interactive reward in the amygdala ROIs was operationalized as the interaction term calculated from the extracted beta values from the bilateral amygdala ROI [Peer Agree – Peer Away] – [Computer Agree – Computer Away]. Age did not significantly predict neural sensitivity to social interaction in the amygdala (β = -0.003, *t*(111)=-0.45, *p*=0.65). Self-reported experience of social-interactive reward in the scanner did not significantly predict neural sensitivity to social interaction in the amygdala (β = 2.15, *t*(111)=0.69, *p*=0.49).

Amygdala response to social-interactive reward marginally predicted desire to interact with the same partner again (β = 112.63, *t*(53)=1.95, *p*=0.06, Supplemental Table 12). When the model was run with the interaction between group and amygdala sensitivity to social-interactive reward included, this interaction term was significant (β = 276.52, *t*(52)=2.42, *p*=0.02, Supplemental Table 13, Supplemental Figure 5). The relation between amygdala sensitivity to social-interactive reward in the TD group was larger and more positive than the relation in the AUT group (TD: *r*_(41)_ = 0.43, *p*=0.004 ASD: *r*_(13)_ = -0.21, *p*=0.45). Amygdala sensitivity to social interaction did not significantly predict interaction quality (β = 0.71, *t*(66)=0.14, *p*=0.89).

*Supplemental Table 12. Regression table for analysis predicting desire to interact with same partner from amygdala sensitivity to social-interactive reward.*

|  | Estimate | Standard Error | *t* | *p* |
| --- | --- | --- | --- | --- |
| Intercept | 46.28 | 51.99 | 0.89 | 0.38 |
| Amygdala activation | 112.63 | 57.63 | 1.95 | 0.06 |
| Group | -7.12 | 16.71 | -0.43 | 0.67 |
| Age at follow-up visit | -0.79 | 3.99 | -0.20 | 0.84 |
| Time between scan and follow-up visit | -4.95 | 7.06 | -0.70 | 0.49 |

*Supplemental Table 13. Regression table for analysis predicting desire to interact with same partner from amygdala sensitivity to social-interactive reward including interaction with group.*

|  | Estimate | Standard Error | *t* | *p* |
| --- | --- | --- | --- | --- |
| Intercept | 28.04 | 50.33 | 0.56 | 0.58 |
| Amygdala activation | -55.45 | 88.67 | -0.63 | 0.53 |
| Group | -10.88 | 16.07 | -0.68 | 0.50 |
| Age at follow-up visit | 0.87 | 3.88 | 0.23 | 0.82 |
| Time between scan and follow-up visit | -5.44 | 6.76 | -0.81 | 0.42 |
| Amgydala * Group | 276.52 | 114.22 | 2.42 | 0.02 |

**

*Supplemental Figure 5*. Amygdala sensitivity to social interaction interacts with group to predict desire to interact with the same partner again during a twenty-five minute face-to-face interaction with a novel peer (*n*=58). Relations were stronger in the TD group (grey) compared to the AUT group (orange).

# fMRIPrep Preprocessing

As requested by the developers of fMRIPrep, the following boilerplate details the preprocessing performed in this manuscript.

Results included in this manuscript come from preprocessing performed using *fMRIPrep* 1.4.1 (Esteban, Markiewicz, et al. (2018); Esteban, Blair, et al. (2018); RRID:SCR_016216), which is based on *Nipype* 1.2.0 (Gorgolewski et al. (2011); Gorgolewski et al. (2018); RRID:SCR_002502).

**Anatomical data preprocessing**

The T1-weighted (T1w) image was corrected for intensity non-uniformity (INU) with N4BiasFieldCorrection (Tustison et al. 2010), distributed with ANTs 2.2.0 (Avants et al. 2008, RRID:SCR_004757), and used as T1w-reference throughout the workflow. The T1w-reference was then skull-stripped with a *Nipype* implementation of the antsBrainExtraction.sh workflow (from ANTs), using MNI152NLin2009cAsym as target template. Brain tissue segmentation of cerebrospinal fluid (CSF), white-matter (WM) and gray-matter (GM) was performed on the brain-extracted T1w using fast (FSL 5.0.9, RRID:SCR_002823, Zhang, Brady, and Smith 2001). Brain surfaces were reconstructed using recon-all (FreeSurfer 6.0.1, RRID:SCR_001847, Dale, Fischl, and Sereno 1999), and the brain mask estimated previously was refined with a custom variation of the method to reconcile ANTs-derived and FreeSurfer-derived segmentations of the cortical gray-matter of Mindboggle (RRID:SCR_002438, Klein et al. 2017). Volume-based spatial normalization to three standard spaces (MNIPediatricAsym, MNI152NLin2009cAsym, MNI152NLin6Asym) was performed through nonlinear registration with antsRegistration (ANTs 2.2.0), using brain-extracted versions of both T1w reference and the T1w template. The following templates were selected for spatial normalization: *MNI’s unbiased standard MRI template for pediatric data from the 4.5 to 18.5y age range* [Fonov et al. (2011), RRID:SCR_008796; TemplateFlow ID: MNIPediatricAsym], *ICBM 152 Nonlinear Asymmetrical template version 2009c* [Fonov et al. (2009), RRID:SCR_008796; TemplateFlow ID: MNI152NLin2009cAsym], *FSL’s MNI ICBM 152 non-linear 6th Generation Asymmetric Average Brain Stereotaxic Registration Model* [Evans et al. (2012), RRID:SCR_002823; TemplateFlow ID: MNI152NLin6Asym].

**Functional data preprocessing**

For each of the 4 BOLD runs found per subject (across all tasks and sessions), the following preprocessing was performed. First, a reference volume and its skull-stripped version were generated using a custom methodology of *fMRIPrep*. A deformation field to correct for susceptibility distortions was estimated based on *fMRIPrep*’s *fieldmap-less* approach. The deformation field is that resulting from co-registering the BOLD reference to the same-subject T1w-reference with its intensity inverted (Wang et al. 2017; Huntenburg 2014). Registration is performed with antsRegistration (ANTs 2.2.0), and the process regularized by constraining deformation to be nonzero only along the phase-encoding direction, and modulated with an average fieldmap template (Treiber et al. 2016). Based on the estimated susceptibility distortion, an unwarped BOLD reference was calculated for a more accurate co-registration with the anatomical reference. The BOLD reference was then co-registered to the T1w reference using bbregister (FreeSurfer) which implements boundary-based registration (Greve and Fischl 2009). Co-registration was configured with nine degrees of freedom to account for distortions remaining in the BOLD reference. Head-motion parameters with respect to the BOLD reference (transformation matrices, and six corresponding rotation and translation parameters) are estimated before any spatiotemporal filtering using mcflirt (FSL 5.0.9, Jenkinson et al. 2002). BOLD runs were slice-time corrected using 3dTshift from AFNI 20160207 (Cox and Hyde 1997, RRID:SCR_005927). The BOLD time-series, were resampled to surfaces on the following spaces: *fsaverage5*. The BOLD time-series (including slice-timing correction when applied) were resampled onto their original, native space by applying a single, composite transform to correct for head-motion and susceptibility distortions. These resampled BOLD time-series will be referred to as *preprocessed BOLD in original space*, or just *preprocessed BOLD*. The BOLD time-series were resampled into several standard spaces, correspondingly generating the following *spatially-normalized, preprocessed BOLD runs*: MNIPediatricAsym, MNI152NLin2009cAsym, MNI152NLin6Asym. First, a reference volume and its skull-stripped version were generated using a custom methodology of *fMRIPrep*. Automatic removal of motion artifacts using independent component analysis (ICA-AROMA, Pruim et al. 2015) was performed on the *preprocessed BOLD on MNI space* time-series after removal of non-steady state volumes and spatial smoothing with an isotropic, Gaussian kernel of 6mm FWHM (full-width half-maximum). Corresponding “non-aggresively” denoised runs were produced after such smoothing. Additionally, the “aggressive” noise-regressors were collected and placed in the corresponding confounds file. Several confounding time-series were calculated based on the *preprocessed BOLD*: framewise displacement (FD), DVARS and three region-wise global signals. FD and DVARS are calculated for each functional run, both using their implementations in *Nipype* (following the definitions by Power et al. 2014). The three global signals are extracted within the CSF, the WM, and the whole-brain masks. Additionally, a set of physiological regressors were extracted to allow for component-based noise correction (*CompCor*, Behzadi et al. 2007). Principal components are estimated after high-pass filtering the *preprocessed BOLD* time-series (using a discrete cosine filter with 128s cut-off) for the two *CompCor* variants: temporal (tCompCor) and anatomical (aCompCor). tCompCor components are then calculated from the top 5% variable voxels within a mask covering the subcortical regions. This subcortical mask is obtained by heavily eroding the brain mask, which ensures it does not include cortical GM regions. For aCompCor, components are calculated within the intersection of the aforementioned mask and the union of CSF and WM masks calculated in T1w space, after their projection to the native space of each functional run (using the inverse BOLD-to-T1w transformation). Components are also calculated separately within the WM and CSF masks. For each CompCor decomposition, the *k* components with the largest singular values are retained, such that the retained components’ time series are sufficient to explain 50 percent of variance across the nuisance mask (CSF, WM, combined, or temporal). The remaining components are dropped from consideration. The head-motion estimates calculated in the correction step were also placed within the corresponding confounds file. The confound time series derived from head motion estimates and global signals were expanded with the inclusion of temporal derivatives and quadratic terms for each (Satterthwaite et al. 2013). Frames that exceeded a threshold of 0.5 mm FD or 1.5 standardised DVARS were annotated as motion outliers. All resamplings can be performed with *a single interpolation step* by composing all the pertinent transformations (i.e. head-motion transform matrices, susceptibility distortion correction when available, and co-registrations to anatomical and output spaces). Gridded (volumetric) resamplings were performed using antsApplyTransforms (ANTs), configured with Lanczos interpolation to minimize the smoothing effects of other kernels (Lanczos 1964). Non-gridded (surface) resamplings were performed using mri_vol2surf (FreeSurfer).

Many internal operations of *fMRIPrep* use *Nilearn* 0.5.2 (Abraham et al. 2014, RRID:SCR_001362), mostly within the functional processing workflow. For more details of the pipeline, see the section corresponding to workflows in fMRIPrep’s documentation.

# Post-Test

The full text of the post-test is provided below. The factor structure for the reward-related items on the post-test was determined as follows. For the 114 participants that contributed useable neural data, a principal components analysis was performed on a subset of post-test items (highlighted in yellow) that captured reward-related constructs to determine the number of factors to extract. Based on Kaiser’s rule, four components were selected with eigenvalues above 1, explaining 63% of the variance. Principal axis factoring was performed on four factors with oblimin rotation. The loadings from this factor analysis are provided in Supplemental Table 14, and loadings greater than 0.4 are bolded. Principal components analysis and principal axis factoring were performed in R using the psych package (Revelle, 2021).

Factor 1 was interpreted to reflect an underlying construct of wanting and liking chatting with the peer. The items that loaded strongly on factor 1 are bolded in the full post-test below. The raw scores from these items were summed into a post-test composite of self-reported wanting and liking chatting with the peer (“Post-test peer enjoyment composite”), which was used to evaluate group differences in self-reported enjoyment of the peer in the scanner task (Figure 2C) and to predict heterogeneity in VS response (Figure 6).

*Supplemental Table 14. Loadings for principal axis factoring of scan post-test with oblimin rotation*

| Question | Factor 1 | Factor 2 | Factor 3 | Factor 4 |
| --- | --- | --- | --- | --- |
| (Pre-scan) 1. How much are you interested in chatting with_____? | **0.64** | 0.12 | 0.10 | -0.11 |
| (Pre-scan) 2. How much do you think you would like ______ in real life? | **0.68** | -0.02 | 0.01 | -0.09 |
| 1. How much did you like chatting with ______? | **0.66** | 0.01 | 0.06 | 0.05 |
| 3. How much do you think you'd like _______in real life? | **0.80** | -0.02 | 0.04 | -0.01 |
| 4. How much did you want to see his/her answer to your question? | **0.51** | -0.08 | 0.24 | -0.14 |
| 5. How did you feel when she/he agreed with your answer? | **0.75** | -0.02 | -0.10 | 0.05 |
| 6. How did you feel when she/he disagreed with your answer? | 0.00 | -0.02 | **0.91** | 0.02 |
| 7. How did you feel when he/she was away and didn't respond? | -0.02 | 0.04 | 0.15 | **0.50** |
| 9. How much do you think ______ would want to be your real-life friend? | **0.71** | -0.06 | -0.12 | 0.07 |
| 12. How much did you like it when you were just answering the computer? | -0.07 | **0.86** | 0.00 | -0.01 |
| 14. How much did you want to see if the computer matched your answer? | 0.35 | 0.38 | -0.01 | -0.08 |
| 15. How did you feel when your answer matched the random answer? | 0.34 | **0.46** | -0.07 | 0.17 |
| 16. How did you feel when your answer did not match the random answer? | 0.00 | 0.27 | 0.39 | 0.24 |
| 17. How did you feel when the computer was disconnected? | -0.02 | -0.02 | 0.01 | **0.74** |

Full Text of Post-test:

Before scan:

1. **How much are you interested in chatting with_____? (1 = not at all, to 5 = a lot)**
2. **How much do you think you would like ______ in real life? (1 = not at all, 5= a lot)**

After scan:

1. **How much did you like chatting with ______? (1 = not at all, to 5 = a lot)**
2. Sometimes it can be hard to pay attention when you're playing games. How much did you pay really, really close attention answering a question when he/she was the one you were chatting with? (1 = not at all, to 5 = a lot)
3. **How much do you think you'd like _______in real life? (1 = not at all, to 5 = a lot)**
4. **How much did you want to see his/her answer to your question? (1 = not at all, to 5 = a lot)**
5. **How did you feel when she/he agreed with your answer? (1 = very bad, to 5 = very good)**
6. How did you feel when she/he disagreed with your answer? (1 = very bad, to 5 = very good)
7. How did you feel when he/she was away and didn't respond? (1 = very bad, to 5 = very good)
8. What did you like the most? (Answered, away, the same)
9. **How much do you think ______ would want to be your real-life friend? (1 = not at all, to 5 = a lot)**
10. Tell me about what kind of person you think ______ is.
11. Why do you think _____ was away for some questions?
12. How much did you like it when you were just answering the computer? (1 = not at all, to 5 = a lot)
13. Sometimes it can be hard to pay attention when you're playing games. How much did you pay really, really close attention answering a question when you were chatting with the computer? (1 = not at all, to 5 = a lot)
14. How much did you want to see if the computer matched your answer? (1 = not at all, to 5 = a lot)
15. How did you feel when your answer matched the random answer? (1 = very bad, to 5 = very good)
16. How did you feel when your answer did not match the random answer? (1 = very bad, to 5 = very good)
17. How did you feel when the computer was disconnected? (1 = very bad, to 5 = very good)
18. What did you like the most? (Connected, Disconnected, Same)
19. Did you like chatting with a person or the computer more? (Person, Computer)
20. Did you pay more attention when it was a person or the computer? (Person, Computer)
21. When you were connected to the computer, did anyone see your answer? (yes, no)
22. When the other person was playing the maze, did they still see your answer? (yes, no)
23. Please move the slider to show how much more you liked chatting with either the person or the computer (or somewhere in the middle). (range: -100 to 100)
24. Please move the slider to show how much more you paid attention when it was either the person or the computer (or somewhere in the middle). (range: -100 to 100)
25. How well did you get to know ______? (1 = Not well at all, to 5 = Really well)
26. How much did _______ share about him/herself? (1 = Nothing to 5 = A lot)
27. How well do you understand how ______ thinks or feels? (1 = Not well at all, to 5 = Really well)
28. How well do you understand what ____ likes or dislikes? (1 = Not well at all, to 5 = Really well)
29. How much would you want to share personal information about yourself with _______? (1 = Nothing to 5 = A lot)
30. Were there any particular questions that were fun to answer or hard to answer?
31. Was _______ a real person? (yes, no)
    1. Why do you think that?
32. Do you think there was more to this game than we told you about? (yes, no)
    1. If yes, what?
33. Is there anything else you want to tell us about the chat task?

# Social Interaction Slider Question

If you came back for a future visit that involves interacting with another child, we could either match you with the same partner as before or a different partner. Please tell us your preference using the slider below. The more you move the slider to the left, the more strongly you feel about wanting the same partner. The more you move the slider to the right, the more strongly you feel about wanting a different partner. If you don’t feel strongly either way, move the slider to somewhere in the middle.

References

Abraham, Alexandre, Fabian Pedregosa, Michael Eickenberg, Philippe Gervais, Andreas Mueller, Jean Kossaifi, Alexandre Gramfort, Bertrand Thirion, and Gael Varoquaux. 2014. “Machine Learning for Neuroimaging with Scikit-Learn.” *Frontiers in Neuroinformatics* 8. <https://doi.org/10.3389/fninf.2014.00014>.

Avants, B.B., C.L. Epstein, M. Grossman, and J.C. Gee. 2008. “Symmetric Diffeomorphic Image Registration with Cross-Correlation: Evaluating Automated Labeling of Elderly and Neurodegenerative Brain.” *Medical Image Analysis* 12 (1): 26–41. <https://doi.org/10.1016/j.media.2007.06.004>.

Behzadi, Yashar, Khaled Restom, Joy Liau, and Thomas T. Liu. 2007. “A Component Based Noise Correction Method (CompCor) for BOLD and Perfusion Based fMRI.” *NeuroImage* 37 (1): 90–101. <https://doi.org/10.1016/j.neuroimage.2007.04.042>.

Cox, Robert W., and James S. Hyde. 1997. “Software Tools for Analysis and Visualization of fMRI Data.” *NMR in Biomedicine* 10 (4-5): 171–78. [https://doi.org/10.1002/(SICI)1099-1492(199706/08)10:4/5<171::AID-NBM453>3.0.CO;2-L](https://doi.org/10.1002/(SICI)1099-1492(199706/08)10:4/5%3c171::AID-NBM453%3e3.0.CO;2-L).

Dale, Anders M., Bruce Fischl, and Martin I. Sereno. 1999. “Cortical Surface-Based Analysis: I. Segmentation and Surface Reconstruction.” *NeuroImage* 9 (2): 179–94. <https://doi.org/10.1006/nimg.1998.0395>.

Egger, H. L., Pine, D. S., Nelson, E., Leibenluft, E., Ernst, M., Towbin, K. E., & Angold, A. (2011). The NIMH Child Emotional Faces Picture Set (NIMH‐ChEFS): a new set of children's facial emotion stimuli. *International journal of methods in psychiatric research*, *20*(3), 145-156. <https://doi.org/10.1002/mpr.343>.

Esteban, Oscar, Ross Blair, Christopher J. Markiewicz, Shoshana L. Berleant, Craig Moodie, Feilong Ma, Ayse Ilkay Isik, et al. 2018. “FMRIPrep.” *Software*. Zenodo. <https://doi.org/10.5281/zenodo.852659>.

Esteban, Oscar, Christopher Markiewicz, Ross W Blair, Craig Moodie, Ayse Ilkay Isik, Asier Erramuzpe Aliaga, James Kent, et al. 2018. “fMRIPrep: A Robust Preprocessing Pipeline for Functional MRI.” *Nature Methods*. <https://doi.org/10.1038/s41592-018-0235-4>.

Evans, AC, AL Janke, DL Collins, and S Baillet. 2012. “Brain Templates and Atlases.” *NeuroImage* 62 (2): 911–22. <https://doi.org/10.1016/j.neuroimage.2012.01.024>.

Fonov, VS, AC Evans, K Botteron, CR Almli, RC McKinstry, and DL Collins. 2011. “Unbiased Average Age-Appropriate Atlases for Pediatric Studies.” *NeuroImage* 54 (1), 313-327. <http://doi.org/10.1016/j.neuroimage.2010.07.033>.

Fonov, VS, AC Evans, RC McKinstry, CR Almli, and DL Collins. 2009. “Unbiased Nonlinear Average Age-Appropriate Brain Templates from Birth to Adulthood.” *NeuroImage* 47, Supplement 1: S102. <https://doi.org/10.1016/S1053-8119(09)70884-5>.

Gorgolewski, K., C. D. Burns, C. Madison, D. Clark, Y. O. Halchenko, M. L. Waskom, and S. Ghosh. 2011. “Nipype: A Flexible, Lightweight and Extensible Neuroimaging Data Processing Framework in Python.” *Frontiers in Neuroinformatics* 5: 13. <https://doi.org/10.3389/fninf.2011.00013>.

Gorgolewski, Krzysztof J., Oscar Esteban, Christopher J. Markiewicz, Erik Ziegler, David Gage Ellis, Michael Philipp Notter, Dorota Jarecka, et al. 2018. “Nipype.” *Software*. Zenodo. <https://doi.org/10.5281/zenodo.596855>.

Greve, Douglas N, and Bruce Fischl. 2009. “Accurate and Robust Brain Image Alignment Using Boundary-Based Registration.” *NeuroImage* 48 (1): 63–72. <https://doi.org/10.1016/j.neuroimage.2009.06.060>.

Huntenburg, Julia M. 2014. “Evaluating Nonlinear Coregistration of BOLD EPI and T1w Images.” Master’s Thesis, Berlin: Freie Universität. <http://hdl.handle.net/11858/00-001M-0000-002B-1CB5-A>.

Jenkinson, Mark, Peter Bannister, Michael Brady, and Stephen Smith. 2002. “Improved Optimization for the Robust and Accurate Linear Registration and Motion Correction of Brain Images.” *NeuroImage* 17 (2): 825–41. <https://doi.org/10.1006/nimg.2002.1132>.

Klein, Arno, Satrajit S. Ghosh, Forrest S. Bao, Joachim Giard, Yrjö Häme, Eliezer Stavsky, Noah Lee, et al. 2017. “Mindboggling Morphometry of Human Brains.” *PLOS Computational Biology* 13 (2): e1005350. <https://doi.org/10.1371/journal.pcbi.1005350>.

Lanczos, C. 1964. “Evaluation of Noisy Data.” *Journal of the Society for Industrial and Applied Mathematics Series B Numerical Analysis* 1 (1): 76–85. <https://doi.org/10.1137/0701007>.

Power, Jonathan D., Anish Mitra, Timothy O. Laumann, Abraham Z. Snyder, Bradley L. Schlaggar, and Steven E. Petersen. 2014. “Methods to Detect, Characterize, and Remove Motion Artifact in Resting State fMRI.” *NeuroImage* 84 (Supplement C): 320–41. <https://doi.org/10.1016/j.neuroimage.2013.08.048>.

Pruim, Raimon H. R., Maarten Mennes, Daan van Rooij, Alberto Llera, Jan K. Buitelaar, and Christian F. Beckmann. 2015. “ICA-AROMA: A Robust ICA-Based Strategy for Removing Motion Artifacts from fMRI Data.” *NeuroImage* 112 (Supplement C): 267–77. <https://doi.org/10.1016/j.neuroimage.2015.02.064>.

Revelle W (2021). *psych: Procedures for Psychological, Psychometric, and Personality Research*. Northwestern University, Evanston, Illinois. R package version 2.1.9, [https://CRAN.R-project.org/package=psych](https://cran.r-project.org/package=psych).

Satterthwaite, Theodore D., Mark A. Elliott, Raphael T. Gerraty, Kosha Ruparel, James Loughead, Monica E. Calkins, Simon B. Eickhoff, et al. 2013. “An improved framework for confound regression and filtering for control of motion artifact in the preprocessing of resting-state functional connectivity data.” *NeuroImage* 64 (1): 240–56. <https://doi.org/10.1016/j.neuroimage.2012.08.052>.

Treiber, Jeffrey Mark, Nathan S. White, Tyler Christian Steed, Hauke Bartsch, Dominic Holland, Nikdokht Farid, Carrie R. McDonald, Bob S. Carter, Anders Martin Dale, and Clark C. Chen. 2016. “Characterization and Correction of Geometric Distortions in 814 Diffusion Weighted Images.” *PLOS ONE* 11 (3): e0152472. <https://doi.org/10.1371/journal.pone.0152472>.

Tustison, N. J., B. B. Avants, P. A. Cook, Y. Zheng, A. Egan, P. A. Yushkevich, and J. C. Gee. 2010. “N4ITK: Improved N3 Bias Correction.” *IEEE Transactions on Medical Imaging* 29 (6): 1310–20. <https://doi.org/10.1109/TMI.2010.2046908>.

Wang, Sijia, Daniel J. Peterson, J. C. Gatenby, Wenbin Li, Thomas J. Grabowski, and Tara M. Madhyastha. 2017. “Evaluation of Field Map and Nonlinear Registration Methods for Correction of Susceptibility Artifacts in Diffusion MRI.” *Frontiers in Neuroinformatics* 11. <https://doi.org/10.3389/fninf.2017.00017>.

Zhang, Y., M. Brady, and S. Smith. 2001. “Segmentation of Brain MR Images Through a Hidden Markov Random Field Model and the Expectation-Maximization Algorithm.” *IEEE Transactions on Medical Imaging* 20 (1): 45–57. <https://doi.org/10.1109/42.906424>.
